# Redox Storm in the Tumor Microenvironment (ΤΜΕ): Multifaceted Redox Injury and Immunometabolic Reprogramming Induced by Immune Checkpoint Blockade in B16F10 Melanoma: An Ultrastructural Study of the Peritumoral Interface

**DOI:** 10.3390/cells15191760

**Published:** 2026-09-27

**Authors:** Vasileios Alexandros Karakousis, Antonia Sioga, Ioannis S. Vizirianakis, Isaak Kesisoglou, Christophoros S. Kosmidis, Leonidas Pavlidis, Theodora Papamitsou

**Affiliations:** 1 School of Medicine, Faculty of Health Sciences, Aristotle University of Thessaloniki, 54124 Thessaloniki, Greece; ikesis@auth.gr (I.K.); kosmidisc@auth.gr (C.S.K.); 2 Laboratory of Histology-Embryology, School of Medicine, Faculty of Health Sciences, Aristotle University of Thessaloniki, 54124 Thessaloniki, Greece; sioga@auth.gr (A.S.); thpapami@auth.gr (T.P.); 3 Department of Plastic Surgery, School of Medicine, Faculty of Health Sciences, Aristotle University of Thessaloniki, Papageorgiou General Hospital, 56403 Thessaloniki, Greece; pavlidisl@auth.gr; 4 Laboratory of Pharmacology, School of Pharmacy, Faculty of Health Sciences, Aristotle University of Thessaloniki, 54124 Thessaloniki, Greece; ivizir@pharm.auth.gr; 5 Department of Health Sciences, School of Life and Health Sciences, University of Nicosia, 1700 Nicosia, Cyprus; 6 Department of Surgery, European Interbalkan Medical Center, 57001 Thessaloniki, Greece

**Keywords:** melanoma, tumor microenvironment, immune checkpoint blockade, transmission electron microscopy, redox storm, ferroptosis, mitochondrial cristolysis, lipid droplets, efferocytosis, metabolic reprogramming

## Abstract

**Highlights:**

**What are the main findings?**

- Transmission electron microscopy provides evidence of a sequential “redox storm” cascade in the peritumoral tumor microenvironment, characterized by T-cell reactivation, mitochondrial cristolysis, lipid droplet accumulation, and efferocytosis.
- Quantitative morphometric analysis at Day 30, performed for comparative purposes, showed that anti-CTLA-4 monotherapy was associated with the highest cell death area (*p* = 0.005), whereas combination therapy was linked to reduced lipid droplet area (*p* = 0.013) and increased foamy macrophage-like cell counts (*p* = 0.010).

**What are the implications of the main findings?**

- This study provides in vivo ultrastructural evidence supporting the “redox storm” model of immune checkpoint blockade, bridging established biochemical mechanisms with tangible structural biology.
- The findings highlight potential redox and metabolic nodes for disrupting immunosuppressive loops, providing a basis for the rational design of next-generation combination immunotherapies.

**Abstract:**

Immune checkpoint blockade (ICB) has revolutionized melanoma treatment, yet the in vivo ultrastructural consequences within the tumor microenvironment (TME) remain poorly characterized. This study investigates the morphological alterations induced by ICB at the peritumoral interface through a “redox storm” paradigm. B16F10 melanoma-bearing mice were treated with anti-CTLA-4, anti-PD-1, or their combination, and evaluated by transmission electron microscopy (TEM) at Days 20, 30, and 40; quantitative morphometric analyses were performed for comparative purposes. This design enabled the assessment of ultrastructural changes from initial immune activation to late-stage remodeling, linking early cytotoxic events with subsequent stromal adaptations. TEM provided evidence of a sequential redox cascade characterized by lymphocyte reactivation, mitochondrial cristolysis, lipid droplet accumulation, foamy macrophage-like cells, efferocytosis-driven M2-like polarization, and metabolic plasticity in surviving tumor cells. Specifically, combination therapy was associated with marked cytolysis and extensive stromal remodeling, while anti-PD-1 appeared to promote prominent cholesterol crystal formation and lipotoxicity. Morphometric analyses supported these qualitative findings, indicating significant alterations in lipid droplet area and cell death area across treatment groups. This ultrastructural dissection provides morphological evidence for the “redox storm” and offers a framework to understand ICB efficacy, resistance, and dynamic TME remodeling, ultimately providing a basis for targeted combination strategies.

## 1. Introduction

### 1.1. Melanoma: Epidemiology, Molecular Drivers, and Clinical Presentation

Melanoma is a highly lethal and aggressive form of skin cancer, accounting for the majority of skin-cancer-related deaths despite comprising only ~4% of cutaneous malignancies, with a rapidly rising global incidence and a male predominance [1,2]. Environmental factors (intermittent, intense ultraviolet (UV) radiation) and host factors (fair skin, numerous nevi, and family history) influence susceptibility to the disease [1,3,4]. Melanomagenesis is driven by aberrant mitogen-activated protein kinase (MAPK) pathway activation, most commonly through mutually exclusive mutations in v-Raf murine sarcoma viral oncogene homolog B (BRAF) (V600E) and neuroblastoma RAS viral oncogene homolog (NRAS), combined with inactivation of tumor suppressors such as cyclin-dependent kinase inhibitor 2A (CDKN2A), phosphatase and tensin homolog (PTEN), and tumor protein p53 (TP53) [1,5]. Clinically, melanoma presents as a changing pigmented lesion and is evaluated using the Asymmetry, Border irregularity, Color variegation, Diameter > 6 mm, and Evolution (ABCDE) criteria, with dermoscopy aiding early detection. Major subtypes include superficial spreading, nodular, lentigo maligna, and acral lentiginous melanoma [3,4]. These subtypes can be categorized into cutaneous and non-cutaneous melanoma, which exhibit distinct etiologies and genomic landscapes. Cutaneous melanoma, including chronically sun-damaged (CSID) and non-chronically sun-damaged (non-CSID) subtypes, is characterized by a high tumor mutational burden and frequent mutations in v-Raf murine sarcoma viral oncogene homolog B (BRAF), neuroblastoma RAS viral oncogene homolog (NRAS), neurofibromin 1 (NF1), and KIT proto-oncogene receptor tyrosine kinase (KIT). In contrast, non-cutaneous melanoma (uveal, mucosal, and acral) presents with a lower mutational burden and distinct genetic drivers, such as G protein subunit alpha q (GNAQ)/G protein subunit alpha 11 (GNA11) in uveal melanoma and KIT in acral and mucosal melanoma, respectively. These molecular differences influence treatment responses to targeted therapies and immunotherapies [6]. Diagnosis is confirmed by excisional biopsy and immunohistochemistry (S100 calcium-binding protein (S100), Human Melanoma Black 45 (HMB45), Melanoma antigen recognized by T cells 1 (Melan-A/MART-1), SRY-related HMG-box 10 (SOX10), and PReferentially Expressed Antigen in Melanoma (PRAME)) while staging follows the American Joint Committee on Cancer (AJCC) 8th edition, integrating Breslow depth, ulceration, nodal involvement, and distant metastasis [1,2,3].

### 1.2. CTLA-4 and PD-1 Checkpoint Inhibition: Mechanisms and Therapeutic Implications

The clinical application of immune checkpoint blockade (ICB) in melanoma began with the approval of ipilimumab (anti-cytotoxic T-lymphocyte-associated protein 4 [CTLA-4]) in 2011, followed by nivolumab (anti-programmed cell death protein 1 [PD-1]) in 2014 [7,8]. Subsequently, additional agents in these classes, including tremelimumab (anti-CTLA-4) and pembrolizumab or toripalimab (anti-PD-1), have expanded the therapeutic armamentarium. CTLA-4 is upregulated on activated T cells and competes with the co-stimulatory receptor cluster of differentiation 28 (CD28) for binding to B7 ligands (cluster of differentiation 80/cluster of differentiation 86 [CD80/CD86]) on antigen-presenting cells, thereby raising the threshold for T-cell activation and maintaining peripheral tolerance [9,10]. Anti-CTLA-4 antibodies (e.g., ipilimumab) disrupt this competition, enhancing CD28-mediated co-stimulation, interleukin-2 (IL-2) production, and effector T-cell expansion; their immunoglobulin G1 (IgG1) Fc domain may contribute to antitumor activity by depleting intratumoral regulatory T cells through Fc gamma receptors (FcγRs) engagement [7,8,10]. In contrast, PD-1 is expressed on activated T, B, and natural killer cells and, upon engagement with its ligands programmed cell death ligand 1/2 (PD-L1/PD-L2), recruits the Src homology 2 domain-containing phosphatase 2 (SHP-2), which dephosphorylates proximal T-cell receptor signaling molecules and drives T-cell exhaustion [7,8,11]. Anti-PD-1 antibodies block this interaction, reinvigorating exhausted cluster of differentiation 8 positive (CD8+) T cells and restoring effector functions such as interferon-gamma (IFN-γ) secretion and cytotoxicity [8,12]. Dual blockade of CTLA-4 and PD-1 (ipilimumab plus nivolumab) yields superior response rates and overall survival compared with either monotherapy, albeit with increased immune-related adverse events, reflecting the complementary nature of these two pathways [7,12]. While combination therapy yields superior clinical outcomes, the precise relative contribution of regulatory T cell (Treg) depletion versus direct T-cell reinvigoration to this synergy remains an area of investigation [8,10]. Quantitative syntheses across preclinical and clinical cohorts have established conserved CD8+ T-cell infiltration and divergent PD-L1 dynamics as key features of immune checkpoint blockade [13].

### 1.3. The Redox and Biochemical Framework of the Peritumoral Interface

The peritumoral compartment has emerged as a clinically pivotal interface, where the density and localization of peritumoral lymphocytes have been shown to correlate significantly with favorable prognostic indicators, including shallower Breslow thickness, lower Clark level, and improved disease-free survival in melanoma patients [14,15]. This clinical relevance underpins our rationale for focusing on the peritumoral interface, as it represents the dynamic boundary where immune activation and stromal remodeling converge to determine therapeutic outcomes. The present study builds upon six proposed mechanistic pathways that link immune checkpoint blockade to oxidative damage and ultrastructural alterations in the tumor microenvironment (TME). Briefly, the pro-oxidant cascade has been suggested to involve: (1) IFN-γ/tumor necrosis factor-alpha (TNF-α)-driven ferroptosis via the suppression of the cystine/glutamate antiporter (xCT)/glutathione peroxidase 4 (GPX4) axis and acyl-CoA synthetase long-chain family member 4 (ACSL4)-mediated lipid peroxidation [16,17]; (2) cardiolipin mono-oxygenation and subsequent mitochondrial cristolysis [18,19]; (3) lipid droplet biogenesis and cluster of differentiation 36 (CD36)-mediated foamy macrophage transformation [20,21]; (4) efferocytosis-driven M2 polarization and NLR family pyrin domain containing 3 (NLRP3) inflammasome activation [22,23]; (5) signal transducer and activator of transcription 6 (STAT6)/peroxisome proliferator-activated receptor gamma (PPARγ)-mediated metabolic reprogramming towards fatty acid oxidation [24,25]; and (6) hypoxia-inducible factor 1-alpha (HIF-1α)-driven metabolic plasticity and oxidative phosphorylation (OXPHOS) upregulation as a resistance mechanism [26,27]. Although these pathways are biochemically distinct, they are presumed to converge on a common “redox storm” whose detailed molecular dissection and correlation with the specific ultrastructural changes observed in our transmission electron microscopy (TEM) analysis are provided in the Discussion section.

### 1.4. B16F10 Syngeneic Model: A Clinically Anchored Platform for Dissecting TME Reprogramming

The B16F10 melanoma model, syngeneic to C57BL/6 mice, is a cornerstone preclinical platform for investigating TME remodeling. Its high metastatic potential and inherent resistance, due to low Major Histocompatibility Complex class I (MHC-I) expression and poor immunogenicity, make it a stringent but clinically relevant system for dissecting the six mechanistic axes of this study [28,29]: the pro-oxidant cascade, mitochondrial damage, lipid droplet accumulation, efferocytosis, M2 polarization, and metabolic plasticity. This rigor enables the distinction between direct oxidative injury (Mechanisms 1–3) and downstream immunosuppressive adaptations (Mechanisms 4–6). Furthermore, its capacity for metabolic adaptation and plasticity under hypoxic and nutrient-deprived stress conditions [30] reinforces its suitability for dissecting mechanisms of metabolic adaptation, while the weak immunogenicity of its neoantigens [31] and the aggressive, recurrent nature of its tumors even following effective immunotherapies [32] underline the translational challenges it presents. Nevertheless, the B16F10 model remains a widely utilized and valuable tool for advancing our understanding of melanoma biology and for evaluating novel therapeutic strategies in a fully immunocompetent setting.

### 1.5. Ultrastructural Dissection of the Peritumoral Interface: Aims and Hypothesis

Despite extensive progress in deciphering the biochemical mechanisms underlying ICB, the in vivo ultrastructural consequences of these therapies within the TME remain largely unexplored. While the molecular pathways driving oxidative stress, lipid peroxidation, and cellular reprogramming have been extensively characterized through genomic, transcriptomic, and proteomic approaches, direct morphological visualization of these events at the peritumoral interface is scarce. To our knowledge, this is the first ultrastructural study to systematically investigate the peritumoral landscape of the B16F10 melanoma TME following anti-CTLA-4 and anti-PD-1 immunotherapy, using TEM to dissect the six interconnected mechanistic axes of the proposed “redox storm”.

We hypothesize that immune checkpoint blockade induces a coordinated, multi-stage redox cascade at the peritumoral interface, manifested by specific, quantifiable ultrastructural signatures. This cascade spans from acute oxidative damage, characterized by mitochondrial cristolysis and lipid droplet accumulation, to downstream immunosuppressive adaptations, including efferocytosis, foamy macrophage polarization, and the metabolic plasticity of surviving tumor cells. Through this structural approach, we aim to bridge established biochemical theories with tangible in vivo evidence, offering a novel morphological framework to better understand ICB efficacy, resistance, and the dynamic remodeling of the TME. This approach identified distinct ultrastructural signatures underlying differential therapeutic efficacy, providing a novel framework for understanding ICB response and resistance. It should be emphasized that the primary aim of this study is to characterize the ultrastructural alterations induced by immune checkpoint blockade; the biochemical mechanisms discussed herein are proposed as a framework for interpreting these morphological findings, and their definitive confirmation will require additional dedicated studies.

## 2. Materials and Methods

### 2.1. Study Design and Ethical Compliance

This study was an analytical, experimental, interventional laboratory investigation conducted on laboratory mice. The study aimed to evaluate the ultrastructural changes in the tumor microenvironment of melanoma following the administration of immunotherapeutic agents. For this study, six to eight-week-old male C57BL/6 mice expressing both human PD-1 and human CTLA-4 receptors (C57BL/6-Pdcd1^tm1(PDCD1)^ Ctla4^tm1(CTLA4)^) were utilized, weighing approximately 28 g at the time of tumor inoculation (Day 0), maintained under specific pathogen-free conditions in the institutional animal facility. The mice were housed in a temperature-controlled environment with a 12-h light/dark cycle and were provided with standard chow and water ad libitum. All animal procedures were performed in accordance with the guidelines of the Institutional Animal Care and Use Committee (IACUC) and national regulations for the care and use of laboratory animals. The complete documentation of the protocol’s licensing and compliance in accordance with the applicable standards is provided in Supplementary File S1.

All experimental procedures were conducted in strict accordance with the guidelines of the European Directive 2010/63/EU, as transposed into national legislation (Presidential Decree 56/2013). The study protocol received ethical approval from the Bioethics and Ethics Committee of the Medical School, Aristotle University of Thessaloniki (Approval No. 64/2025) and a formal experimental license from the Directorate of Veterinary Medicine of the Region of Central Macedonia (License No. 500963[1745]).

In line with the PREPARE guidelines [33], the study was designed to ensure robust preparation prior to initiation. A comprehensive literature search was performed to establish the scientific rationale for the chosen model (B16F10 melanoma in C57BL/6 mice) and to define clear study objectives. All personnel involved in the experiments had the necessary education and training in animal handling, anesthesia, and surgical procedures. A risk assessment was conducted prior to the study, covering health risks, waste disposal, and decontamination protocols. The animals’ housing and husbandry conditions were evaluated and optimized, including a 7-day acclimatization period, environmental enrichment (nesting material, tunnels), and ad libitum access to food and water under standard laboratory conditions (12 h light/dark cycle, 22 ± 2 °C, humidity 50 ± 10%). Humane endpoints were defined objectively and pre-specified to minimize suffering, based on criteria such as >20% weight loss, immobility, tumor ulceration exceeding 10% of body surface, as well as the onset of severe clinical signs including neurological deficits, respiratory distress, or persistent diarrhea. Following tumor cell inoculation, mice were monitored every 24 h for body weight, behavior, and tumor palpation. After the initiation of immunotherapy and following skin biopsies, animals were monitored every 12 h for adverse events (e.g., diarrhea, lethargy) and for post-operative pain, wound healing, and potential infection.

The final manuscript adheres to the ARRIVE 2.0 guidelines [34] to ensure full transparency in reporting. A completed ARRIVE 2.0 checklist, detailing the precise location of each reported item within the manuscript, has been provided as a Supplementary File. The study design included four experimental groups: a Control group and three treatment groups (Anti-CTLA-4, Anti-PD-1, and their Combination), with clear definition of the experimental unit (the individual animal). Randomization of animals to treatment groups was performed using a computer-generated random sequence (Microsoft Excel, Microsoft Corporation, Redmond, WA, USA; Microsoft 365, RAND function), and the investigator conducting the histological and ultrastructural analyses was blinded to the treatment allocation. The primary outcome measures were defined as the extent of tumor regression, mitochondrial damage, and immune cell infiltration, as assessed by ultrastructural and histological analysis. Statistical methods were pre-specified and are detailed in the corresponding section. Inclusion and exclusion criteria were established a priori, and no animals were excluded from the final analysis. The number of animals used (*n* = 32 in total) is explicitly reported, in accordance with the 3Rs principles.

In alignment with the 3Rs (Replacement, Reduction, Refinement) principles, the Reduction was achieved by utilizing the number of animals per group indicated by a priori power analysis, ensuring statistical validity while minimizing animal usage. Refinement was implemented through the use of multimodal analgesia (buprenorphine, xylocaine), temporary single-housing post-biopsy to prevent wound infection and aggressive behavior, and enhanced post-operative monitoring. A mandatory retrospective assessment conducted one year after the study (June 2026) by the Veterinary Directorate confirmed the successful implementation of these measures and the overall achievement of the study’s objectives, with no unanticipated adverse events or protocol deviations.

### 2.2. Cell Culture and Tumor Model

For the in vivo tumor model, in vitro cultures of B16-F10 cells were initiated on Day-10. The highly metastatic murine melanoma cell line B16-F10 (American Type Culture Collection, ATCC^®^ CRL-6475™; Manassas, VA, USA), originally isolated from C57BL/6 mice, was used. Cells were cultured in Dulbecco’s Modified Eagle’s Medium (DMEM; ATCC^®^ 30-2002™) supplemented with 10% (*v*/*v*) heat-inactivated fetal bovine serum (FBS; ATCC^®^ 30-2025™) and 100 µg/mL penicillin–streptomycin (Gibco™ Pen-Strep, Thermo Fisher Scientific, Waltham, MA, USA). The cultures were maintained at 37 °C in a humidified atmosphere containing 5% (*v*/*v*) CO_2_. For routine subculturing, cells were washed with Dulbecco’s phosphate-buffered saline without calcium or magnesium (DPBS; ATCC^®^ 30-2200™) and detached using 0.25% (*w*/*v*) trypsin–EDTA solution (ATCC^®^ 30-2101™) upon reaching approximately 80% confluence, typically every 1–2 days. Cells were used at passages lower than 25 to maintain genetic stability and reproducibility. For long-term preservation, cells were cryopreserved in complete DMEM containing 10% (*v*/*v*) dimethyl sulfoxide (DMSO; ATCC^®^ 4-X™) and stored at −80 °C until use.

On Day 0, melanoma was induced by subcutaneous injection of 1 × 10^5^ B16-F10 cells suspended in 0.1 mL of phosphate-buffered saline (PBS) into the right flank of the mice [29,31]. Treatment with ipilimumab (YERVOY^®^; Bristol-Myers Squibb, Princeton, NJ, USA) and nivolumab (OPDIVO^®^; Bristol-Myers Squibb, Princeton, NJ, USA) was initiated on Day 10, when the tumors reached a suitable volume for therapeutic intervention, consistent with the established growth kinetics of the B16-F10 model [29]. For consistency with the existing literature and to facilitate direct comparison across studies, these therapeutic agents will hereafter be uniformly referred to as anti-CTLA-4 and anti-PD-1.

### 2.3. Treatment Protocol

To assess the effects of immune checkpoint blockade on the tumor microenvironment, mice were randomly assigned (as detailed in Section 2.1) into four experimental groups (*n* = 8 per group):

- Anti-CTLA-4 monotherapy: Administered intraperitoneally (i.p.) at a dose of 10 mg/kg once weekly.
- Anti-PD-1 monotherapy: Administered i.p. at a dose of 5 mg/kg twice weekly.
- Combination therapy: Administered i.p. with 10 mg/kg anti-CTLA-4 weekly and 5 mg/kg anti-PD-1 twice weekly.
- Control group: Administered i.p. with an equivalent volume of vehicle (PBS) on the same schedule.

Treatments were initiated on Day 10 post-inoculation, when tumors were established, and continued over a period of 30 days (until Day 40), according to the established protocol [29]. The doses and schedules were selected based on clinically relevant and preclinical pharmacokinetic/pharmacodynamic data to ensure optimal receptor saturation and therapeutic efficacy in the murine model.

### 2.4. Pharmacokinetic and Pharmacodynamic Analysis for the Murine Dosing Regimen

To ensure clinically relevant and pharmacologically effective immune checkpoint blockade, the doses, routes, and schedules of anti-CTLA-4 and anti-PD-1 monoclonal antibodies were selected based on a comprehensive analysis of existing preclinical and clinical pharmacokinetic (PK) and pharmacodynamic (PD) data. The dosing regimen employed in this study was designed to achieve therapeutic exposures while accounting for the species-specific differences in antibody metabolism and target binding.

#### 2.4.1. Dose Selection and Justification

The dose of anti-PD-1 (5 mg/kg) was selected based on its demonstrated efficacy in syngeneic murine tumor models. The anti-tumor activity of anti-PD-1 is dose-dependent, with significant tumor growth inhibition observed at doses as low as 1 mg/kg, but optimal and consistent responses achieved at 5 mg/kg, as reported in the MC38 colon carcinoma model [35,36]. Furthermore, PK studies in mice have shown that a dose of 5 mg/kg is sufficient to saturate the PD-1 receptor in peripheral blood and tumor tissue, as demonstrated by receptor occupancy assays [36,37]. This saturation is critical for achieving the maximum pharmacological effect of PD-1 blockade.

For anti-CTLA-4, the dose of 10 mg/kg was chosen based on established preclinical protocols that demonstrate robust anti-tumor efficacy. Studies using surrogate anti-CTLA-4 antibodies in mouse models have shown that doses in this range are necessary to achieve effective intratumoral T-cell modulation and tumor regression [38,39]. In particular, the Fc-effector function of anti-CTLA-4 antibodies, which is important for their therapeutic activity in mice, has been shown to be optimal at this dose level [38]. Furthermore, the differential dosing frequency between anti-PD-1 and anti-CTLA-4 is not driven by differences in systemic clearance, but rather by the prolonged local retention of anti-CTLA-4 within the tumor microenvironment [39]. Collectively, these preclinical data confirm that the selected doses of anti-CTLA-4 (10 mg/kg) and anti-PD-1 (5 mg/kg) ensure optimal receptor saturation and therapeutic efficacy in the syngeneic B16F10 melanoma model, providing a robust foundation for the experimental design of this study.

#### 2.4.2. Rationale for Dosing Frequency

The difference in administration frequency between anti-CTLA-4 (weekly) and anti-PD-1 (twice weekly) is driven by their distinct PK profiles in mice. Anti-PD-1 exhibits a faster clearance in mice compared to humans, with a terminal half-life of ~45–50 h reported in wild-type C57BL/6 mice [40]. Consequently, a more frequent dosing schedule (e.g., every 3–4 days or twice weekly) is required to maintain sustained therapeutic concentrations, as illustrated in Figure 1 and supported by the dosing regimens used in mouse models [35,41,42]. Notably, the profiles shown in Figure 1 are in silico simulations based on published murine pharmacokinetic parameters and are not derived from experimental measurements performed in this study.

In contrast, anti-CTLA-4 antibodies exhibit a significantly longer half-life in mice, with a reported terminal half-life of 3.25 days for the anti-CTLA-4 surrogate 9H10 [43]. Furthermore, anti-CTLA-4 antibodies have been shown to exhibit prolonged retention within the tumor microenvironment following administration. A study using [89Zr]-labeled anti-CTLA-4 has demonstrated that approximately 6–8% of the injected dose remains in the tumor 7 days after a single intratumoral injection, providing sustained local exposure [39]. This prolonged tumor retention, combined with the well-established weekly dosing regimen used in the B16F10 preclinical model [44], supports a once-weekly administration schedule for anti-CTLA-4.

#### 2.4.3. Route of Administration: Intraperitoneal Delivery

Intraperitoneal (i.p.) injection was selected as the route of administration for both antibodies. This route is widely accepted and validated in murine models of immunotherapy. i.p. injection is a standard and well-tolerated method for delivering monoclonal antibodies in mice, as evidenced by its extensive use in numerous preclinical efficacy studies [35,41,44,45]. In the MC38 tumor model, i.p. administration of nivolumab at 10 mg/kg consistently produced robust anti-tumor effects, confirming the efficacy of this route [35]. Furthermore, in MC38 tumor-bearing mice, i.p. administration of the anti-PD-1 surrogate muDX400 (a pembrolizumab analogue) at 5 mg/kg achieved plasma concentrations (~55 µg/mL) sufficient for PD-1 receptor saturation and yielded dose-dependent antitumor activity, further validating this route for efficacy studies [36]. Consistent with these findings, i.p. administration of anti-CTLA-4 (100 µg) and anti-PD-1 (200 µg) in CT26 and 4T1 syngeneic models resulted in significant tumor growth inhibition and reduced lung metastasis (for anti-CTLA-4), while being well tolerated, reinforcing the i.p. route as a highly effective and broadly applicable delivery method for immune checkpoint blockade in mice [46].

#### 2.4.4. Comparison with Human Dosing and Rationale for Interspecies Differences

The dosing regimen used in this study reflects the significant interspecies differences in antibody pharmacokinetics (PK) between mice and humans. In humans, ipilimumab is administered at 3 mg/kg every 3 weeks, and nivolumab at 240 mg every 2 weeks (or 480 mg every 4 weeks), as per the approved Summary of Product Characteristics (SmPC) [47,48]. However, the PK profile of monoclonal antibodies in mice is characterized by a faster clearance and a much shorter half-life. Using nivolumab, a striking discrepancy between the murine half-life (~2 days) and the human half-life (~25 days) was demonstrated [40], further underscoring the vast interspecies differences in immunoglobulin G (IgG) catabolism.

To achieve a similar area under the concentration–time curve (AUC) and receptor saturation as observed in humans, higher doses and more frequent administration are required in mice [36]. For example, PK modeling has shown that a dose of 5 mg/kg in mice achieves high receptor occupancy, a key determinant of efficacy, which was used to inform the selection of clinical doses in humans, thus ensuring the clinical relevance of the murine studies [36,49]. Importantly, the mice systemic pharmacokinetics (e.g., clearance, distribution, and metabolism) remain fundamentally murine. These are governed by mouse-specific hepatic, renal, and neonatal Fc receptor (FcRn)-mediated clearance mechanisms. Consequently, interspecies scaling (based on body weight and organ function) is required to achieve human-equivalent therapeutic exposures in mice. This explains why higher doses and more frequent administration schedules are necessary in our murine model compared to the standard human regimen.

In conclusion, the chosen regimen of 10 mg/kg anti-CTLA-4 weekly and 5 mg/kg anti-PD-1 twice weekly, administered intraperitoneally (i.p.), is a well-established, scientifically justified protocol that ensures therapeutic efficacy, accounts for murine-specific PK/PD characteristics, and maintains clinical relevance.

### 2.5. Sample Collection and Processing

Skin biopsies were collected on Days 20 and 30 post-inoculation using a 0.5 cm punch biopsy followed by suturing. At the experimental endpoint (Day 40), mice were euthanized via CO_2_ inhalation followed by cervical dislocation. Primary skin tumors were harvested and processed for Transmission Electron Microscopy (TEM) analysis as part of the present ultrastructural study. Furthermore, additional skin and secondary organs samples (lungs, liver, brain, and bones) were collected for future histological and immunohistochemical analyses (Hematoxylin & Eosin, Masson’s trichrome, and immunostaining for CD8 (Cluster of differentiation 8) and α-smooth muscle actin (α-SMA)), which will be presented in a separate manuscript. Additionally, whole blood was collected via intracardiac puncture at the time of euthanasia using ethylenediaminetetraacetic acid (EDTA)-coated tubes. Plasma was separated by centrifugation (1500× *g* for 10 min at 4 °C) and stored at −80 °C. The detailed biochemical analysis of these plasma samples will be presented in a dedicated, separate manuscript, as the current study focuses exclusively on the ultrastructural (TEM) evidence of oxidative damage and stromal remodeling.

### 2.6. Transmission Electron Microscopy (TEM) Sample Preparation and Imaging

For transmission electron microscopy (TEM), primary skin tumor tissue samples were initially cut into pieces smaller than 1 cm^3^. Fixation was carried out by immersing the samples in a 3% glutaraldehyde solution for 1 h, followed by rinsing in phosphate buffer for 10 min. The samples were then post-fixed in a 1% osmium tetroxide (OsO_4_) solution for 1–1.5 h at 4 °C, rinsed again with phosphate buffer for 10 min, and washed with bi-distilled water for another 10 min. En bloc staining was performed with 1% uranyl acetate solution for 12–14 h overnight at 4 °C, followed by a final wash with bi-distilled water for 10 min. Dehydration was achieved through a graded ethanol series: 30°, 50°, 70°, 96°, and 100° (5 min each). Infiltration and embedding were performed using a 1:1 mixture of Epon and absolute ethanol for 10 min, followed by centrifugation in pure Epon for 20 min at 2500 rpm, and finally polymerization at 65–70 °C for 24 h. For the ultrastructural examination, the resin-embedded tissue blocks were initially trimmed using a Leica^®^ EM TRIM2 (Leica Microsystems, Wetzlar, Germany). Prior to ultrathin sectioning, semi-thin sections were obtained and stained with toluidine blue to precisely identify and select the peritumoral target regions of interest. Subsequently, ultrathin sections (70–90 nm) were cut with a Leica^®^ EM UC6 ultramicrotome (Leica Microsystems, Wetzlar, Germany) utilizing glass knives prepared on a Reichert^®^ Knifemaker (Reichert Technologies, Depew, NY, USA). The sections were mounted onto 200-mesh copper grids and subsequently stained with Reynolds’ lead citrate to enhance contrast. Finally, they were examined with a JEOL^®^ JEM-1011 (JEOL Ltd., Tokyo, Japan) transmission electron microscope operating at an accelerating voltage of 80 kV. Digital images were acquired using a Gatan^®^ Erlangshen ES500W (Gatan, Inc., Pleasanton, CA, USA) charge-coupled device (CCD) camera (Model 782).

### 2.7. Quantitative Morphometric Analysis and Sampling Strategy

Although the overall experimental cohort consisted of 8 mice per group (*n* = 8), the quantitative morphometric analysis by TEM was conducted on a randomly selected, representative subset of 3 mice per group (*n* = 3) due to the labor-intensive and resource-demanding nature of ultrastructural sample preparation and analysis. More specifically, for the quantitative morphometric analysis of the 2nd biopsy (Day 30), a total of 10 random fields of view (FOVs) were selected per mouse. This approach was applied across the four experimental groups, resulting in a total of 120 FOVs analyzed. The sample size was justified based on established methodological standards in ultrastructural morphometry; specifically, it was demonstrated that the analysis of 30 cells per animal is sufficient to obtain statistically robust and representative data [50]. Recently, this approach was further corroborated in an ultrastructural analysis of neuronal mitochondria and synapses, utilizing *n* = 3 biological replicates combined with multiple technical measurements per condition to achieve statistically robust data [51]. Furthermore, the use of *n* = 3 biological replicates for quantitative ultrastructural analysis was validated in a TEM study of nuclear pore size changes following excitotoxic injury in the neonatal brain [52]. Structural alterations were effectively quantified using linear electron density profiles and non-parametric statistical tests, providing a robust precedent for the sampling strategy and statistical approach employed in our study. Furthermore, the applicability of *n* = 3 biological replicates for quantitative TEM studies of myelin vacuolization was demonstrated in the cuprizone model [53], where multiple random images per animal (*n* = 10 images per mouse) and non-parametric statistical tests (Mann–Whitney and Kruskal–Wallis) were utilized, further reinforcing the validity of the sampling strategy and statistical approach adopted in our study. Supporting this strategy, 10 random FOVs per condition were utilized for quantitative assessment in a scanning electron microscopy-based assay [54], confirming that the selection of a defined number of random fields is a well-established practice for reliable ultrastructural morphometry. It should be emphasized that the cited ultrastructural studies [50,51,52,53,54], although conducted in different experimental settings and assessing different parameters, similarly employed *n* = 3 animals per group due to the inherent challenges of TEM-based morphometry. Additionally, no separate power analysis was performed for this *n* = 3 morphometric subset; the a priori power analysis described in Section 2.1 was designed to determine the sample size of the overall in vivo cohort (*n* = 8 per group; 32 animals in total). Therefore, the quantitative morphometric data presented herein are intended exclusively for comparative purposes and do not constitute the basis of our conclusions.

### 2.8. Statistical Analysis

Image analysis was performed using Fiji (ImageJ, National Institutes of Health, Bethesda, MD, USA) [55] and statistical analyses were conducted using JASP (Version 0.96.0.0 [56]; JASP Team, Amsterdam, The Netherlands). For each FOV, four morphometric parameters were selected for quantitative analysis: Tumor-Infiltrating Lymphocytes (TILs), Foamy Macrophage-like Cells (Mφ), Lipid Droplet (LD) Area, and Cell Death Area. These parameters were selected given that they directly reflect key mechanisms of action of immune checkpoint blockade (i.e., T-cell infiltration, lipid-mediated oxidative stress, and cell death), and owing to their consistent presence in the vast majority of the acquired fields of view, which allowed robust quantification. The resulting values were averaged per mouse, and the mean value for each mouse was subsequently used as the independent data point for the statistical comparisons (*n* = 3 per group). To minimize potential overestimation of a single death modality, measurements of necrotic and apoptotic areas were pooled and collectively termed “cell death” throughout the manuscript, figures, and tables. All data were tested for normality using the Shapiro–Wilk test [57]. For comparisons among the four experimental groups, a one-way analysis of variance (ANOVA) [58] followed by Tukey’s post hoc test [59] was applied for normally distributed data, whereas the Kruskal–Wallis test [60] followed by Dunn’s multiple comparison test [61] was used for non-parametric data. All data are presented as mean ± standard error of the mean (SEM). A *p*-value of < 0.05 was considered statistically significant for all analyses. The number of biological replicates (*n*) is specified in the respective sections. A schematic summary of the complete study design, including the experimental timeline, treatment groups, and analytical workflow, is presented in Figure 2.

## 3. Results

### 3.1. Light Microscopic Evaluation of the Peritumoral Zone

The tumor growth patterns differed across the experimental groups: the Control group exhibited continuous tumor growth, whereas the Anti-CTLA-4 group showed a steady reduction in tumor volume, the Anti-PD-1 group showed a smaller reduction, and the Combination group showed the most pronounced reduction; the detailed tumor volume values for the three mice per group used in the TEM analysis are provided in Supplementary Table S1. Semi-thin sections were stained with toluidine blue, a metachromatic dye that highlights acidic tissue components and provides excellent contrast for evaluating tissue architecture [62], to precisely identify and select the peritumoral target regions of interest, where the immunotherapeutic agents exert their primary effects, as shown in Figure 3. The original, entire images, as captured from the light microscope, are provided in Supplementary File S2. Examination under a light microscope at ×10 magnification showed a progressive spatial evolution of the tumor mass across the experimental groups. In the control group, the tumor exhibited continuous growth and vertical expansion from the initial subcutaneous injection site toward the epidermal layer. In contrast, the treated groups showed a progressive reduction in tumor mass over time. Notably, in the combination group, considerable regression with replacement by loose, fibrotic connective tissue was observed by the final time point. Although toluidine blue staining is not diagnostic for specific cell types, it provided a visualization of the tumor architecture and its interaction with the surrounding stromal and epidermal structures, highlighting the differential therapeutic responses among the groups.

### 3.2. Ultrastructural Characterization of the Peritumoral Interface Across Treatment Groups

Representative TEM images were acquired from the peritumoral interface of B16F10 melanomas across the four treatment groups (Control, Anti-CTLA-4, Anti-PD-1, and Combination). The images were selected to illustrate the full spectrum of ultrastructural alterations underlying the six mechanistic axes analyzed in this study and are presented in Figure 4, Figure 5, Figure 6 and Figure 7, and are also included in Supplementary File S3. Specifically, the panels depict: (i) the pro-oxidant cascade and ferroptotic features (Mechanism 1); (ii) mitochondrial cristolysis and damage (Mechanism 2); (iii) lipid droplet dynamics and foamy macrophage-like cell formation (Mechanisms 3 and 5); (iv) efferocytosis and apoptotic clearance (Mechanism 4); and (v) the metabolic plasticity and resistance features of surviving tumor cells (Mechanism 6). A detailed mechanistic interpretation of these findings, integrated with the corresponding biochemical pathways, is provided in the Discussion section.

A comprehensive summary of the main light microscopic and ultrastructural findings across the four experimental groups is presented in Figure 8. This schematic consolidates the key observations from Figure 3, Figure 4, Figure 5, Figure 6 and Figure 7, providing a side-by-side comparison of the tumor evolution and the corresponding ultrastructural alterations induced by anti-CTLA-4, anti-PD-1, and their combination.

### 3.3. Morphometric Analysis of the Peritumoral Microenvironment Indicates Distinct Immune and Metabolic Alterations Following Immune Checkpoint Blockade

To quantify the ultrastructural changes induced by immune checkpoint blockade, we performed a systematic morphometric analysis on TEM images obtained from the peritumoral interface of B16F10 melanomas. Four parameters were assessed: Tumor-Infiltrating Lymphocytes (TILs), Foamy Macrophage-like Cells (Foamy Mφ), Lipid Droplet Area (LD Area), and Cell Death Area. Measurements were conducted blindly by two independent researchers using Fiji (ImageJ, version 1.54p; National Institutes of Health, Bethesda, MD, USA), and any discrepancies were resolved by a senior third investigator. Due to the small sample size and the non-normal distribution of some parameters, both parametric (ANOVA) and non-parametric (Kruskal–Wallis) tests were performed to ensure robust statistical assessment, followed by Dunn’s post hoc comparisons where appropriate. The complete raw dataset for all morphometric parameters is available in Supplementary Table S4. Detailed descriptive statistics, including mean, standard deviation, minimum, and maximum values for all parameters, are provided in Supplementary Table S4. All morphometric data presented in this section, Table 1, and Figure 9 were derived from the 2nd biopsy (Day 30) and are therefore representative of a single time point.

*Tumor-infiltrating lymphocytes (TILs).* TIL density did not differ significantly among the groups (ANOVA, *p* = 0.470; Kruskal–Wallis, *p* = 0.228). A numerical increase was observed in the Combination group; however, it did not reach statistical significance and was accompanied by notable inter-individual variation. This heterogeneity may reflect the individual-specific nature of the immune response to dual checkpoint blockade.

*Foamy macrophage-like cells (Foamy Mφ).* The number of foamy macrophage-like cells differed significantly among groups (ANOVA, *p* = 0.039; Kruskal–Wallis, *p* = 0.027). Dunn’s post hoc analysis revealed that Combination group significantly increased Foamy Mφ counts compared to Control group (*p* = 0.010), and Anti-PD-1 group also showed a significant increase versus Control group (*p* = 0.019). This suggests that immune checkpoint blockade is associated with features consistent with macrophage activation and phagocytic clearance of apoptotic debris.

*Lipid droplet area (LD Area).* Additionally, LD Area exhibited a highly significant overall effect (ANOVA, *p* = 0.003). Post hoc Dunn’s tests confirmed that Combination therapy significantly reduced lipid droplet accumulation compared to Control group (*p* = 0.013), while Anti-CTLA-4 monotherapy also led to a significant reduction relative to Control group (*p* = 0.024). These findings suggest that combination blockade may prevent lipid storage, possibly through metabolic collapse and extensive cell death.

*Cell death area (Cell Death Area).* Cell death area was significantly affected by treatment (ANOVA, *p* = 0.007; Kruskal–Wallis, *p* = 0.045). Dunn’s tests showed that Anti-CTLA-4 group exhibited a markedly higher cell death area compared to Control group (*p* = 0.005; Bonferroni-adjusted *p* = 0.027). Although a numerical increase in cell death area was observed in the Combination group, it did not reach statistical significance in pairwise comparison versus the Control group.

A summary of the morphometric results is presented in Table 1. The full statistical outputs, including ANOVA, Kruskal–Wallis, and Dunn’s post hoc comparisons for all parameters, are presented in Supplementary Table S4.

Collectively, the morphometric analysis indicated distinct treatment-dependent alterations in the peritumoral microenvironment. The distribution of TILs showed a notable, albeit heterogeneous, increase in the Combination group compared to all other groups, although this did not reach statistical significance (Figure 9A). Foamy Mφ counts were significantly elevated in Combination and Anti-PD-1 groups (Figure 9B). Moreover, LD Area was significantly reduced following Combination and Anti-CTLA-4 treatment compared to controls (Figure 9C), while Cell Death Area was most pronounced in the Anti-CTLA-4 group (Figure 9D). These data suggest that immune checkpoint blockade is associated with marked alterations in the metabolic and immunological landscape of the melanoma tumor microenvironment.

It should be noted that the study is subject to inherent limitations associated with TEM-based morphometry. The labor-intensive nature of the technique and the need for multiple random fields of view per animal restricted the sample size to *n* = 3 per group. Despite this small size, this number is consistent with the number of biological replicates used in analogous ultrastructural studies [50,51,52,53]. Additionally, manual tracing of the morphometric features in Fiji introduces a risk of measurement error. Nevertheless, the analysis was conducted under strictly controlled conditions and, acknowledging these limitations, serves as a comparative assessment of the ultrastructural changes induced by the different immunotherapeutic regimens.

## 4. Discussion

### 4.1. A Clinically Relevant Pro-Oxidant Cascade May Link Immune Checkpoint Blockade to Ultrastructural Damage

Although the detailed biochemical quantification of systemic oxidative stress markers is beyond the scope of the present ultrastructural analysis and will be reported elsewhere, our TEM findings of mitochondrial cristolysis and lipid droplet accumulation are consistent with the morphological signature of the redox storm, as extensively documented in the literature. Specifically, biochemical evidence has demonstrated that anti-PD-1 immunotherapy exerts its antitumor effects through ferroptosis, as liproxstatin-1 reversed its efficacy [70]. This was further supported by evidence that mefloquine synergizes with IFN-γ to activate the Signal Transducer and Activator of Transcription 1 (STAT1)-Interferon Regulatory Factor 1 (IRF1)-Lysophosphatidylcholine Acyltransferase 3 (LPCAT3) axis, enhancing lipid peroxidation and CD8+ T-cell infiltration [71]. Conversely, xCT inhibition was shown to trigger an immunosuppressive counter-response via exosomal PD-L1 and M2 macrophage polarization [72]. In a testicular toxicity model, it was demonstrated that anti-PD-1 disrupts the Nuclear Factor Erythroid 2-Related Factor 2 (NRF2)-Solute Carrier Family 7 Member 11 (SLC7A11)-Glutathione Peroxidase 4 (GPX4) axis, leading to ferroptosis, thus providing mechanistic evidence for this pathway in response to anti-PD-1 therapy [73]. Additionally, evidence indicates that vinburnine-induced Reactive Oxygen Species (ROS) activates the p38 Mitogen-Activated Protein Kinase (P38)/Activating Transcription Factor 3 (ATF3)/Interleukin 24 (IL-24) axis, enhancing CD8+ T-cell cytotoxicity [74]. Crucially, this pro-oxidant framework extends to CTLA-4 blockade. Low baseline glutathione (GSH) has been linked to poor overall survival in ipilimumab-treated patients [75], while elevated Lactate Dehydrogenase (LDH) and its early increase were shown to predict resistance to ipilimumab [76]. These findings were corroborated in combination regimens [77], confirming that oxidative burden is a clinically relevant determinant of therapeutic outcome. Consequently, our TEM findings are consistent with a morphological correlate of this established biochemical cascade.

### 4.2. A Sequential Redox Cascade: From Mitochondrial Injury to Microenvironmental Reprogramming

The ultrastructural hallmarks of oxidative injury documented in our TEM analysis, ranging from mitochondrial cristolysis and lipid droplet accumulation to foamy macrophage-like cell transformation and stromal destruction, do not appear to be isolated, random events. Rather, they appear to represent a temporally and mechanistically interconnected cascade that seems to initiate with the lymphocyte reactivation-driven pro-oxidant cascade and ferroptotic cell death (Mechanism 1), which could exert a direct oxidative assault on the mitochondrial apparatus (Mechanism 2) and might propagate sequentially through the lipid metabolic landscape (Mechanism 3), is proposed to engage the efferocytic and immunosuppressive reprogramming of macrophages (Mechanisms 4 and 5), and may culminate in the metabolic plasticity that could permit residual tumor cells to survive and resist ongoing immune pressure (Mechanism 6). To systematically dissect this stepwise progression, we will discuss each of these six intertwined axes, linking our TEM findings to the specific molecular pathways that are proposed to define the evolution of the redox storm from immunological synapse activation to microenvironmental adaptation and therapy resistance.

#### 4.2.1. Mechanism 1: The Pro-Oxidant Cascade and Ferroptotic Cell Death as the Ultrastructural Hallmark of Lymphocyte Reactivation

The pro-oxidant cascade seems to be initiated by lymphocyte reactivation. The efficacy of immune checkpoint blockade (ICB) relies on disinhibition of exhausted CD8^+^ T lymphocytes, restoring their secretion of interferon-γ (IFN-γ) and tumor necrosis factor-α (TNF-α) [78]. In our TEM analysis, dense lymphocytic infiltrates, uropods loaded with electron-dense granules, and polarization toward B16F10 cells are consistent with a morphological correlate of this reactivation. Vectorial secretion of cytotoxic molecules is orchestrated by reorientation of the microtubule-organizing center (MTOC) and Golgi complex toward the immunological synapse [66]. Our observations suggest that this synaptic architecture may serve as a structural basis for oxidative payload delivery.

It is important to note that IFN-γ exhibits a dual role in melanoma immunotherapy, functioning not only as a key mediator of anti-tumor immunity but also, under certain conditions, as a promoter of immune evasion and tumor progression. This duality depends on the duration of exposure, the tumor microenvironment context, and the activation state of the signaling pathways involved. While acute IFN-γ signaling enhances antigen presentation and cytotoxic T-cell function, chronic IFN-γ exposure has been associated with adaptive immune resistance, including upregulation of PD-L1 and indoleamine 2,3-dioxygenase (IDO), and the promotion of a dedifferentiated, immune-evasive melanoma phenotype [79]. Notably, recent evidence from preclinical melanoma models suggests that loss of IFN-γ sensitivity by tumor cells can paradoxically enhance anti-tumor immunity through remodeling of the myeloid compartment and increased infiltration of antigen-specific CD8^+^ T cells [80]. This complexity underscores the context-dependent nature of IFN-γ signaling within the tumor microenvironment and should be considered when interpreting the pro-oxidant cascade described here.

In this cascade, the first arm is driven by IFN-γ. IFN-γ engages the Janus Kinase (JAK)/STAT1 axis to suppress the cystine/glutamate antiporter system Xc^−^ (SLC7A11 and Solute Carrier Family 3 Member 2 (SLC3A2)), blocking cystine import [16,17,81,82,83,84]. This depletes intracellular cysteine, the rate-limiting precursor for glutathione (GSH) biosynthesis, inactivating glutathione peroxidase 4 (GPX4), the sole enzyme detoxifying membrane phospholipid hydroperoxides. Simultaneously, IFN-γ via STAT1/IRF1 upregulates acyl-CoA synthetase long-chain family member 4 (ACSL4), which esterifies arachidonic and adrenic acids into membrane phospholipids, enriching the bilayer with peroxidation-prone polyunsaturated fatty acid (PUFA) substrates [16,17]. This dual action primes the melanoma cell membrane for oxidative damage. The swollen mitochondria, disrupted cristae, and electron-lucent matrix we document seem to represent the morphological correlate of this GPX4 failure and subsequent lipid peroxidation.

The second arm is mediated by TNF-α and iron-driven Fenton chemistry. TNF-α promotes ferritin degradation, releasing redox-active ferrous iron (Fe^2+^) into the labile iron pool [85]. Fe^2+^ fuels the Fenton reaction (Fe^2+^ + H_2_O_2_ → Fe^3+^ + •OH + OH^−^), generating the hydroxyl radical (•OH) that initiates lipid peroxidation [86,87]. The hydrogen peroxide substrate can be supplied by immune cells via nicotinamide adenine dinucleotide phosphate (NADPH) oxidase 2 (NOX2), linking the immunological synapse to chemical execution of the tumor cell [86]. Lipid droplets, particularly the osmiophilic-bounded droplets in the Anti-PD-1 and Combination groups, is compatible with sequestration of peroxidized lipids, an adaptive buffer that could be overwhelmed when Fenton flux exceeds antioxidant capacity [17,20]. The cascade is further sustained by NADPH oxidase 1 (NOX1) and NOX2. The NOX1-dipeptidyl peptidase 4 (DPP4) complex generates superoxide anion (O_2_•^−^) [88], which propagates lipid peroxidation and ferroptosis, particularly when p53 is inactivated.

Convergence of these arms, diminished antioxidant capacity (IFN-γ-driven GSH depletion) and elevated pro-oxidant iron/ROS generation (TNF-α-driven ferritin degradation, Fenton chemistry, NOX activation), is likely to constitute the “redox storm” overwhelming the tumor cell’s antioxidant defenses [16,17,89]. The terminal event could be ferroptotic cell death, characterized by lethal lipid peroxides, mitochondrial damage, and plasma membrane rupture. Dying tumor cells release damage-associated molecular patterns (DAMPs, such as high mobility group box 1 (HMGB1) and adenosine triphosphate (ATP)) that propagate anti-tumor immunity [16].

Delivery of these effector molecules is highly coordinated at the ultrastructural level, as recently elucidated [67,90]. The electron-dense granules we observe might correspond to single-core and multi-core granules (SCGs and MCGs). MCGs, which release exosomes enriched in cluster of differentiation 63 (CD63) and pro-apoptotic proteins in a Munc13-4-dependent manner, exhibit superior cytotoxic activity and deliver perforin and granzyme B directly into the synaptic cleft [67]. In parallel, T-cell-derived small extracellular vesicles (T-sEVs) carry IFN-γ, TNF-α, and cytotoxic molecules, spreading the oxidative assault beyond the synaptic contact [90].

Collectively, our TEM findings appear to provide a morphological sequence of this cascade. They imply that the redox storm may be a tangible, pharmacologically induced event whose consequences scale with lymphocyte reactivation intensity, with the strongest manifestations in the Combination group where IFN-γ and TNF-α synergy is most pronounced.

#### 4.2.2. Mechanism 2: Mitochondrial Damage, Lipid Droplet Accumulation, and the Ultrastructural Hallmarks of the Redox Storm

The pro-oxidant cascade described in Mechanism 1 appears to converge upon the mitochondrion. Melanoma cells are particularly vulnerable due to heightened mitochondrial activity that increases electron leakage and basal superoxide (O_2_•^−^) flux [18,91]. When immune checkpoint blockade unleashes the IFN-γ/TNF-α cytokine storm, which dismantles the glutathione (GSH)/GPX4 shield, this flux may be amplified into a redox crisis [92,93].

Opto-lipidomics has demonstrated that mono-oxygenation of C18:2-containing cardiolipins serves as a pro-apoptotic death signal [19]. We propose that Fenton-derived •OH and NOX-derived superoxide, described in Mechanism 1 [91,92], may similarly target these cardiolipins, potentially leading to cristae disruption and mitochondrial dysfunction. The morphological translation of this injury seems reflected in our TEM images, which show swollen mitochondria with fragmented cristae and an electron-lucent matrix. This phenotype is consistent with the morphological correlate of •OH-driven lipid peroxidation and mitochondrial dysfunction described previously [18,92,93,94], and could parallel the intensity of lymphocyte infiltration.

IFN-γ upregulates Nitric Oxide Synthase 2 (NOS2), generating nitric oxide that collapses the mitochondrial membrane potential, fueling further O_2_•^−^ leakage while stabilizing HIF-1α to rewire metabolism toward glycolysis [95]. Disruption of fatty acid oxidation (FAO) causes accumulation of unoxidized polyunsaturated fatty acids, which undergo ROS-driven peroxidation to generate aldehydes (acrolein) that compromise the inner membrane [96,97].

Cells sequester peroxidized lipids into lipid droplets, abundant in our TEM fields. When Fenton flux exceeds this buffering capacity, peroxidized lipids overwhelm GPX4 defenses, triggering ferroptotic membrane rupture [18,92,93,96]. This damage also acts as a signaling event: oxidative stress activates AMP-activated Protein Kinase (AMPK), tagging PD-L1 for ubiquitin-proteasomal degradation and removing the immune-evasive shield [98].

Aggressive melanoma cells expel structurally compromised mitochondria when the burden exceeds mitophagy. B16F10 cells shift toward a fission-biased state (elevated phospho-dynamin-related protein 1 (DRP1), downregulation of PTEN-induced kinase 1 (PINK1)/Autophagy-related gene 7 (ATG7), upregulation of BCL2 interacting protein 3 (BNIP3)/NIP3-like protein X (NIX)), secreting mitochondria with near-total cristae loss [99]. Our observation of extracellular debris may thus reflect accumulation of these ejected mitochondria, which fuel the inflammatory and immunosuppressive milieu. These expelled mitochondria are structurally and functionally compromised. Their accumulation has been implicated as a potential driver of local inflammation and immune modulation. This escape mechanism suggests a metabolic adaptation: rather than succumbing to oxidative injury, a fraction of melanoma cells may dispose of damaged organelles to evade the redox storm, potentially sustaining therapy-resistant persisters.

This vulnerability is unlikely to be solely an artifact of immunotherapy and may represent an intrinsic property of melanoma cells. Pharmacologically induced oxidative stress, via Signal Transducer and Activator of Transcription (STAT3) suppression, produces identical ultrastructural signatures [100], and similar damage has been reported with complex I inhibition [91,101]. Collectively, our TEM findings offer a morphological framework for this cascade, with the mitochondrion emerging as a central focal point whose damage correlates with dual checkpoint blockade intensity.

#### 4.2.3. Mechanism 3: Lipid Droplet Dynamics, Foamy Macrophage-like Cell Formation, and the Ferroptotic Axis

The pro-oxidant cascade also reshapes the lipid landscape of the tumor microenvironment. Lipid droplets (LDs) are redox-active sentinels that sequester peroxidized lipids, protecting membranes from ferroptotic damage [17,20]. The lipid-rich tumor microenvironment creates a permissive niche for immunosuppression, where lipid accumulation in tumor-associated macrophages (TAMs) and CD8^+^ T cells promotes M2 polarization and effector dysfunction [21,97]. Extrinsic factors, including selenium and specific lipid species, can modulate ferroptotic sensitivity and confer resistance to immune-mediated killing [102]. Our TEM observations of round, gray, membrane-free LDs in B16F10 cells, foamy macrophage-like cells, and tumor-infiltrating lymphocytes (TILs) are consistent with this cytoprotective response.

Ferroptosis execution is biochemically precise. The lethal signal is enzymatic generation of doubly and triply oxygenated phosphatidylethanolamine (PE) species containing arachidonic (C20:4) or adrenic (C22:4) acids [103]. This is orchestrated by ACSL4 (esterifying fatty acids), LPCAT3 (incorporating them into membrane PE), and 15-lipoxygenase (oxygenating PUFA chains) [103]. The resulting PE hydroperoxides (PE-OOH) act as proximate death signals. When GPX4 is functional, these are reduced; under IFN-γ-mediated glutathione depletion (Mechanism 1), PE-OOH species accumulate. Excessive lipid accumulation in CD8^+^ T cells and macrophages has been highlighted to impair effector functions and promote immunosuppression [97]. The prominent LDs we observe appear to represent the morphological consequence of cells sequestering oxidized acyl chains into neutral lipid cores [17,20].

The protective capacity of LDs is limited by diacylglycerol O-acyltransferase 1 (DGAT1). DGAT1 catalyzes triglyceride synthesis, channeling peroxidation-prone PUFAs into inert LDs [104]. When overwhelmed, unsequestered PUFAs are shunted into oxidative pathways, triggering ferroptosis that sensitizes tumors to immune checkpoint blockade [104]. Our TEM detection of LDs alongside shrunken, electron-dense mitochondria with reduced cristae, the hallmark of ferroptosis, suggests that the peritumoral battlefield may be heterogeneous. While LDs represent a protective buffer, their coexistence with ferroptotic mitochondria implies that this buffering capacity has been exceeded in some cells.

The lipid-rich TME imposes an oxidative burden on infiltrating immune cells. CD36-mediated uptake of fatty acids and oxidized lipoproteins by CD8^+^ T cells and TAMs contributes to lipid peroxidation, impairing T-cell effector function [105,106,107]. This renders ICB-reactivated lymphocytes potentially vulnerable to lipid-driven ferroptotic exhaustion, possibly limiting the durability of the antitumor response.

This lipid overload is possibly consequential for macrophages. As efferocytosis increases in the ICB-treated TME, internalization of cholesterol-rich membranes could overwhelm reverse cholesterol transport machinery, leading to cholesterol crystal precipitation within the phagolysosome [108]. In vivo models have shown that cholesterol overload triggers crystal formation and phagolysosomal membrane rupture [109]. By analogy, crystals might pierce the phagolysosomal membrane in the TME, triggering NLRP3 inflammasome activation and caspase-1-dependent IL-1β secretion [108]. Our observation of foamy macrophage-like cells distended with LDs and phagolysosomes, alongside polygonal, electron-lucent crystals, may represent the morphological endpoint of this cholesterol-driven injury, which has the potential to convert beneficial efferocytosis into a maladaptive, pro-inflammatory cascade.

The interplay between CD36-mediated lipid uptake, DGAT1-dependent lipid storage, and ACSL4-driven lipid peroxidation establishes a redox axis linking metabolic vulnerability to immunotherapy outcome [103,104,105]. The prominent LDs we quantify by TEM appear to represent a hallmark of ferroptotic stress [102,104]. Targeting this axis represents a promising strategy to convert immunologically “cold” tumors into “hot” ones and overcome incomplete tumor elimination [104,105,110]. Our data provide morphological support for this lipid-centered redox axis in B16F10 melanoma. Figure 10 provides a schematic summary of Mechanisms 1–3.

#### 4.2.4. Mechanism 4: Efferocytosis, Foamy Macrophage-like Cell Formation, and the Redox-Driven Immunosuppressive Axis

The clearance of apoptotic tumor cells by macrophages, efferocytosis, has been described as a “Janus-faced” gatekeeper of immunity. While essential for tissue homeostasis, in the peritumoral microenvironment it appears to be subverted into a self-perpetuating engine of immunosuppression and immune evasion [23,111]. Our TEM observations of phagocytic cups, cargo-laden phagolysosomes, and foamy macrophage-like cells distended with lipid droplets in the Anti-PD-1 and Combination groups are consistent with an ultrastructural correlate of this active efferocytic program.

The process is initiated by apoptotic B16F10 cells, which broadcast “find-me” signals such as ATP, lysophosphatidylcholine, and sphingosine-1-phosphate, recruiting macrophages [112,113,114]. Macrophages then engage the “eat-me” signal: phosphatidylserine (PS), externalized on the apoptotic cell surface. This externalization has been dissected as a caspase-3/7-dependent event [115], where executioner caspases cleave the flippases ATPase phospholipid transporting 11A/C (ATP11A/C) and activate the scramblase XK-related protein 8 (XKR8), exposing PS. This PS is recognized by a receptor network including Tyro3, Axl, and MerTK receptor tyrosine kinases, T-cell immunoglobulin and mucin domain-containing molecule 4 (TIM-4), and integrins αvβ3/αvβ5, which orchestrate formation of the phagocytic cup we document [23,111,112,113,114].

The mode of cell death seems to influence the receptor system engaged. While apoptotic cells are canonically cleared via the phosphatidylserine (PS)/myeloid-epithelial-reproductive tyrosine kinase (MerTK) axis, wherein externalized PS is recognized by MerTK through bridging molecules such as growth arrest-specific 6 (Gas6) [23,111,112,114,115,116], ferroptotic cells (Mechanism 3) might present a distinct ‘eat-me’ signal: the oxidized phosphatidylethanolamine species SAPE-OOH (1-stearoyl-2-15-hydroperoxyeicosatetraenoic acid (HpETE)-sn-glycero-3-PE). This lipid acts as a ligand for Toll-like receptor 2 (TLR2), engaging a PS-independent clearance pathway. Our observation of organized apoptotic bodies and cytolytic debris suggests that both PS/MerTK and SAPE-OOH/TLR2 axes may be operating simultaneously.

Engulfment of this cargo, rich in cholesterol, oxidized phospholipids, and peroxidized lipids, generates a secondary wave of oxidative stress within the phagolysosome [112,113]. This lipid overload activates nuclear receptors peroxisome proliferator-activated receptor gamma (PPARγ) and liver X receptor alpha (LXRα), which upregulate efferocytic machinery (MerTK, Axl receptor tyrosine kinase (Axl), Gas6), locking the macrophage into a feed-forward loop of engulfment and M2-like polarization [111,112,113,117]. Concurrently, oxidative stress promotes Microtubule-associated protein 1A/1B-light chain 3 (LC3)-associated phagocytosis (LAP), reinforcing the tolerogenic phenotype [117]. This reprogramming toward fatty acid oxidation is likely to contribute to lipid droplet accumulation, consistent with the “foamy” macrophage-like cell phenotype we observe.

A potential redox consequence may be cholesterol crystal precipitation. When reverse cholesterol transport machinery ATP-binding cassette transporter A1/G1 (ABCA1/ABCG1) is overwhelmed, free cholesterol crystallizes into needle-shaped structures within the phagolysosome [108]. These crystals pierce the phagolysosomal membrane, triggering NLRP3 inflammasome assembly, caspase-1 activation, and non-pyroptotic secretion of IL-1β [22,23,108]. This inflammasome axis sustains chronic inflammation and tumor growth [22], while driving secretion of interleukin-10 (IL-10) and transforming growth factor-beta (TGF-β) that suppresses cytotoxic T-cell function [23,111]. The aryl hydrocarbon receptor (AhR)/ALK and LTK ligand 1 (ALKAL1) axis further sustains this MerTK-dependent phenotype, as AhR-driven ALKAL1 has been shown to phosphorylate and activate MerTK [116].

The overwhelming apoptotic burden induced by dual checkpoint blockade can saturate this efferocytic machinery. When clearance capacity is exceeded, apoptotic cells progress to secondary necrosis, releasing immunostimulatory DAMPs (such as HMGB1-nucleosome complexes) that convert tolerogenic death into an immunogenic event [118,119]. Our observation of massive cytolysis and amorphous debris in the Combination group is suggestive of this saturation. Pharmacological inhibition of TAM kinases or blockade of PS recognition disrupts efferocytosis, promoting secondary necrosis and repolarizing macrophages toward a pro-inflammatory M1 phenotype [119]. This suggests that foamy macrophage-like cells may not merely be passive repositories of debris but may also be active participants in a therapeutic paradox: the same efferocytic clearance that removes tumor cells killed by Anti-CTLA-4 and Anti-PD-1 appears to simultaneously seed the next wave of immune escape. Thus, our TEM data offer an ultrastructural framework for targeting the redox-driven efferocytosis axis to potentially disrupt the vicious cycle of immunosuppression.

#### 4.2.5. Mechanism 5: Metabolic Reprogramming of Macrophages and the Redox-Driven M2-like Polarization Axis

The foamy, lipid-laden macrophage-like cells we observe by TEM, particularly in Anti-PD-1 and Combination groups, point to a metabolic reprogramming wherein tumor-associated macrophages (TAMs) are skewed toward an M2-like, immunosuppressive phenotype. This transition is orchestrated by interleukin-4 (IL-4)/interleukin-13 (IL-13)-driven JAK/STAT6 signaling, cooperating with PPARγ and liver X receptor (LXR) to redirect metabolism toward fatty acid oxidation (FAO) [17,24,120,121,122]. This shift fuels secretion of IL-10, TGF-β, and prostaglandin E2 (PGE_2_), while upregulating PD-L1 and depleting arginine and tryptophan via arginase 1 (ARG1) and indoleamine 2,3-dioxygenase (IDO), suppressing CD8^+^ T-cell function [17,24,120,121,123].

PPARγ and LXR promote fatty acid uptake and β-oxidation, sustained by CD36, fostering M2 polarization [24,112,113,123]. This switch is gated by two nodes: the late endosomal/lysosomal adaptor and MAPK and mTOR activator 1 (Lamtor1)-mechanistic target of rapamycin complex 1 (mTORC1) axis, integrating nutrient and cytokine signals to drive LXR-dependent ARG1 and IL-10 expression [124], and the STAT6-mechanistic target of rapamycin complex 2 (mTORC2)-interferon regulatory factor 4 (IRF4) pathway, coordinating the transcriptional program for the FAO switch [25].

Within phagolysosomes, internalization of cholesterol-rich apoptotic debris delivers oxysterol ligands activating LXR. This activation has been shown to induce a dual program [125]: upregulating MerTK to enhance efferocytosis, while promoting a tolerogenic TGF-β/IL-10 signature. Excess cholesterol has been reported to precipitate into flat, rhomboid crystals [69], causing lysosomal destabilization and cathepsin B leakage, triggering NLRP3 inflammasome activation and caspase-1-dependent IL-1β secretion. The cholesterol crystal ghosts offer a structural correlate to this inflammasome-driven cascade, paradoxically sustaining chronic inflammation while reinforcing the M2 state.

Arachidonic acid from phagocytosed cargo fuels cyclooxygenase-2 (COX-2)/microsomal prostaglandin E synthase-1 (mPGES-1)-dependent synthesis of prostaglandin E_2_ (PGE_2_). PGE_2_, via E prostanoid receptors 2 and 4 (EP2/EP4), has been established to amplify the immunosuppressive milieu [126,127] by upregulating PD-L1 and recruiting regulatory T-cells (Tregs) through the mature dendritic cell enriched in immunoregulatory molecules (mregDC)–Treg axis. This creates a loop where macrophages clearing debris become custodians of immune evasion, expressing PD-L1 that exhausts CD8^+^ T cells. Therefore, targeting ROS production or lipid signaling could disrupt this M2 polarization loop, converting immunosuppressive TAMs into pro-inflammatory effectors supporting anti-tumor immunity [120,121].

Thus, the foamy macrophage-like cell captured by TEM might not be merely a passive scavenger, but could represent the endpoint of a metabolic–immunological axis integrating efferocytosis, lipid metabolism, and transcriptional reprogramming. Targeting nodes such as CD36, carnitine palmitoyltransferase 1 (CPT1), COX-2, or Lamtor1–mTORC1 may offer a strategy to disrupt this loop, potentially repolarizing TAMs toward M1-like phenotype and improving immune checkpoint blockade efficacy in B16F10 melanoma.

A notable observation at Day 40 was extensive pathological fibrosis replacing neoplastic tissue. This is consistent with sustained M2-like-polarized macrophage activity. M2-TAMs have been highlighted to participate in tissue remodeling and induce collagen synthesis, while granulin promotes fibrosis [120]. It has been emphasized that TAMs enhance expression and organization of collagen I and VI, contributing to stroma stiffening and a physical barrier [121]. Evidence links M2-TAM activity to collagen deposition, stroma stiffening, and ECM remodeling [120,121]. Therefore, fibrosis may represent the terminal phase, where oxidative insult (Mechanisms 1–3) and efferocytic clearance (Mechanism 4) drive M2-like polarization (Mechanism 5), leading to a fibrotic scar that seals the ‘battlefield’ but might also create a niche for residual tumor cells (Mechanism 6).

#### 4.2.6. Mechanism 6: Metabolic Plasticity, Mitochondrial Reprogramming, and the HIF-1α/PDK–OXPHOS Axis in Therapy Resistance

Melanoma cells exhibit metabolic plasticity governed by HIF-1α (hypoxia-inducible factor 1-alpha) and peroxisome proliferator-activated receptor gamma coactivator 1-alpha (PGC-1α), which promotes oxidative phosphorylation (OXPHOS) and antioxidant defense [26,128,129,130]. Mitochondrial damage we document may act as a redox trigger. Excess superoxide (O_2_•^−^) inhibits Fe^2+^-dependent prolyl hydroxylases (PHDs), stabilizing HIF-1α under normoxia [131,132]. This stabilization might be more than passive, possibly a redox signaling event: HIF-1α upregulates glucose transporters (GLUT1), hexokinase 2 (HK2), and lactate dehydrogenase A (LDHA), shifting to aerobic glycolysis and creating an immunosuppressive, lactate-rich microenvironment impairing CD8^+^ T-cell function.

This shift is reinforced by HIF-1α-mediated upregulation of Pyruvate dehydrogenase kinase 1 (PDK1) [129] and Pyruvate dehydrogenase kinase 3 (PDK3) [27], which inactivate pyruvate dehydrogenase (PDH). This “glycolytic lock” blocks pyruvate entry into the tricarboxylic acid (TCA) cycle, diverting carbon to lactate and suppressing mitochondrial ROS, providing survival advantage [27]. Our TEM observation of cytoplasmic vacuolization, dilated ER, and autolysosomes could reflect metabolic stress, while persistent viability might indicate engagement of this HIF-1α-driven escape program.

To compensate for compromised TCA cycle, melanoma cells engage activating transcription factor 4 (ATF4)-driven integrated stress response (ISR). ATF4 upregulates glutamine transporter SLC1A5 (ASCT2) and glutaminase 1 (GLS1), sustaining TCA anaplerosis [133]. This ATF4-glutamine axis is critical when pyruvate oxidation is blocked by PDK1/PDK3 [27,129], providing carbon and nitrogen for biosynthesis and redox balance. Coexistence of damaged mitochondria with viable tumor cells is consistent with an ultrastructural readout: while electron transport chain is compromised, TCA cycle may continue through glutamine-fuelled anaplerosis, allowing survival.

This metabolic tug-of-war extends to TME, where tumor-driven glucose and glutamine depletion impairs CD8^+^ T-cell function and promotes immunosuppression [134]. This glycolytic phenotype protects melanoma cells but starves effector T cells, creating immune evasion cycle. Upon exposure to immune checkpoint inhibitors, melanoma cells frequently upregulate OXPHOS genes as resistance mechanism. By shifting to mitochondrial respiration via PGC-1α/melanocyte-inducing transcription factor (MITF) axis and engaging MCL1 apoptosis regulator (MCL1), TNF receptor-associated protein 1 (TRAP1), and sirtuin 3 (SIRT3), surviving cells maintain ATP and redox balance despite oxidative assault [26,128,135].

This adaptation is influenced by host mitochondrial genetics. Specific mitochondrial DNA (mtDNA) haplogroups, such as mitochondrial haplogroup T (HG-T), are associated with nivolumab resistance via upregulation of ROS-detoxification pathways (glutamate–cysteine ligase catalytic subunit (GCLC), glutathione peroxidase 3 (GPX3)) in peripheral T cells [136]. This dampens ROS signaling required for CD8^+^ T-cell differentiation, creating PD-1 blockade resistance. Thus, HIF-1α/PDK–glutamine axis [27,129,133] and OXPHOS shift [26,128,135] may represent metabolic checkpoint determining survival, potentially offering targetable vulnerability for combination strategies (e.g., dichloroacetate (DCA) [27], GLS1 inhibitors [133]) with immune checkpoint blockade [134]. Figure 11 provides schematic summary of efferocytosis-driven immunosuppressive reprogramming of macrophages and HIF-1α/ATF4-mediated metabolic plasticity of surviving melanoma cells described in Mechanisms 4–6.

Figure 12 provides a detailed chemical diagram of the redox storm, illustrating the sequential generation of reactive oxygen species (ROS) from NOX1/NOX2 and mitochondria, the Fenton and Haber–Weiss chemistry, and the propagation of lipid peroxidation and mitochondrial damage described in Mechanisms 1–4, culminating in the collapse of the antioxidant system (xCT/GSH/GPX4) and subsequent cell death (Mechanisms 5–6). This schematic is supported by the mechanistic frameworks highlighted in recent studies, including the NOX-driven superoxide production and mitochondrial electron leakage [137], the iron-dependent Fenton chemistry and hydroxyl radical formation [138,139], and the GPX4-mediated lipid peroxidation leading to cell death [137,138,139], along with the broader mitochondrial dysfunction and metabolic plasticity frameworks [140,141].

### 4.3. Synthesis of the Redox Cascade and Therapeutic Perspectives

Collectively, the six mechanistic axes suggest a sequential redox cascade initiating with lymphocyte reactivation and progressing through mitochondrial injury, lipid remodeling, efferocytic immunosuppression, and ultimately tumor metabolic plasticity. This provides a framework for viewing the redox storm as a dynamic multi-stage process where initial oxidative injury (Mechanisms 1 and 2) may elicit cytoprotective responses (Mechanism 3) that, once overwhelmed, may be subverted into immunosuppressive circuits (Mechanisms 4–5), potentially leading surviving cells into metabolic adaptation (Mechanism 6). Future research should focus on dissecting the precise temporal kinetics of this cascade, as the optimal window for therapeutic intervention may vary between the acute oxidative phase and the chronic immunosuppressive phase. United by the common currency of ROS and antioxidant depletion, our TEM findings offer a structural footprint of this coordinated assault. To translate these structural findings into actionable biomarkers, future studies should incorporate spatial transcriptomics and single-cell metabolomics to correlate these specific ultrastructural signatures with the underlying molecular state of individual cells within the peritumoral niche. Therapeutically, this framework points to possible vulnerabilities: targeting NOX or PDK could amplify early cytotoxicity, while inhibiting CD36 or COX-2 may break immunosuppressive loops. Thus, disrupting this self-sustaining redox cycle at multiple nodes may be key to converting the tumor microenvironment from a tolerogenic niche into an immunostimulatory battlefield. Moreover, further investigation is needed to determine the optimal sequencing of these combinatorial interventions, for instance, whether NOX or PDK inhibition should precede or follow CD36/COX-2 blockade to maximize efficacy and minimize toxicity. Preclinical models employing longitudinal sampling and advanced imaging, combined with clinical trials incorporating redox-based patient stratification, could be critical to validate these targets and unravel the dynamic interplay between oxidative injury and immune adaptation, ultimately potentially paving the way for personalized redox-modulating immunotherapies. Future mechanistic and biochemical studies are warranted to validate the proposed pathways and to quantify the oxidative stress markers underlying the observed ultrastructural alterations. More specifically, future validation of these findings in humanized PDX models with a fully reconstituted immune system will be essential for clinical translation. Recent advances, such as patient-autologous thymic organoids that support functional T-cell education in humanized mice bearing melanoma xenografts, provide a promising platform for such validation [142]. Furthermore, all-autologous humanized melanoma models have been shown to recapitulate patient-specific clinical responses to immune checkpoint blockade, offering a clinically relevant system to confirm the translational potential of our observations [143]. In addition to humanized PDX models, indications of the immune contribution to the observed immunometabolic reprogramming can be obtained through comparative studies between immunocompetent and immunodeficient hosts. Consistent with this need, recent studies comparing immunocompetent and immunodeficient hosts have demonstrated that immune competence is required for the anti-tumor effects of redox-modulating interventions in melanoma [144]. Furthermore, loss of tumor-derived immunosuppressive factors has been shown to reduce tumor growth and remodel the immune microenvironment in immunocompetent but not in immunodeficient mice, supporting the value of comparative designs across hosts with differing immune competence [145].

### 4.4. Limitations

The interpretation of the present findings should be considered alongside the specific limitations associated with the experimental model, ultrastructural methodology, and quantitative analysis. The intrinsic limitations of the experimental model represent a primary consideration. The B16F10 syngeneic model, while a cornerstone of preclinical melanoma research, is characterized by low MHC-I expression and poor immunogenicity compared to human melanomas [29]. Consequently, although this model provides a rigorous platform for dissecting tumor microenvironment (TME) remodeling, it does not fully replicate the complex immunobiology, genetic heterogeneity, and clinical progression of human melanoma. Furthermore, the immune environment is fundamentally murine, potentially introducing species-specific differences in cellular cross-talk and systemic pharmacokinetics [28]. A recognized technical limitation inherent to transmission electron microscopy (TEM) involves artifacts created during ultramicrotomy, such as surface scratches or variations in section thickness. These preparation-induced features can occasionally obscure biological structures. To mitigate this, we actively excluded images exhibiting such artifacts during our analysis; images containing them were only retained when these artifacts did not compromise the quality or interpretation of the depicted structures that were essential for the study. Furthermore, regarding the quantitative morphometric analysis, the labor-intensive nature of TEM restricted the sample size to *n* = 3 mice per group. Although this is consistent with established precedent in ultrastructural literature [50,51], it could constrain the power of statistical inference, and future experiments with larger sample sizes will be required to validate these observations. Additionally, manual tracing in Fiji might introduce a risk of measurement error, and the pooling of apoptotic and necrotic areas as “cell death” may obscure the distinct molecular pathways governing these modalities. Moreover, this study relies exclusively on ultrastructural morphological criteria to identify immune cells (e.g., lobulated nuclei, lytic granules, pseudopodia). While TEM provides unmatched resolution of cellular ultrastructure, definitive characterization of specific immune subpopulations (e.g., cluster of differentiation 8 positive (CD8^+^) vs. cluster of differentiation 4 positive (CD4^+^) T cells, classically activated macrophage/pro-inflammatory macrophage (M1) vs. alternatively activated macrophage (M2) macrophages) requires additional complementary techniques, such as immunoelectron microscopy(immuno-EM) or flow cytometry. The absence of these specific markers may restrict our ability to conclusively link distinct morphological phenotypes to their exact molecular identities. Finally, it should be emphasized that the mechanistic pathways discussed in this manuscript are based on established literature and are proposed as an interpretative framework for our ultrastructural findings; they were not directly measured in this study. Therefore, they should be regarded as hypothesis-generating rather than confirmatory, and future dedicated biochemical, molecular, and functional studies will be required to validate them. Despite these considerations, the present study adheres to the rigorous standards of electron microscopy and provides a detailed ultrastructural picture of the tumor microenvironment following therapeutic intervention.

## 5. Conclusions

In conclusion, this study offers an ultrastructural dissection of the peritumoral interface following immune checkpoint blockade, providing evidence of the multifaceted “redox storm” associated with melanoma regression. Our TEM analysis suggests a sequential cascade, from lymphocyte reactivation and mitochondrial cristolysis to lipid droplet accumulation, efferocytosis, and metabolic plasticity that may culminate in distinct therapeutic signatures. While anti-CTLA-4 and anti-PD-1 monotherapies were associated with significant oxidative injury, combination therapy was accompanied by marked cytolysis and extensive stromal fibrosis, whereas anti-PD-1 monotherapy was linked to prominent cholesterol crystal formation and lipotoxicity. By bridging established biochemical pathways with tangible in vivo morphology, this framework might offer a novel understanding of ICB efficacy and resistance. Ultimately, by integrating redox biology with ultrastructural evidence, this work may provide a conceptual foundation for targeting the dynamic interplay between oxidative injury and immune adaptation, potentially offering a roadmap to convert the tumor microenvironment from a tolerogenic niche into an immunostimulatory battlefield. Nevertheless, it should be acknowledged that while this study provides a morphological framework linking ultrastructural alterations to established biochemical pathways, definitive confirmation of the proposed mechanisms will require further dedicated biochemical and mechanistic investigations.

## Figures and Tables

**Figure 1:**
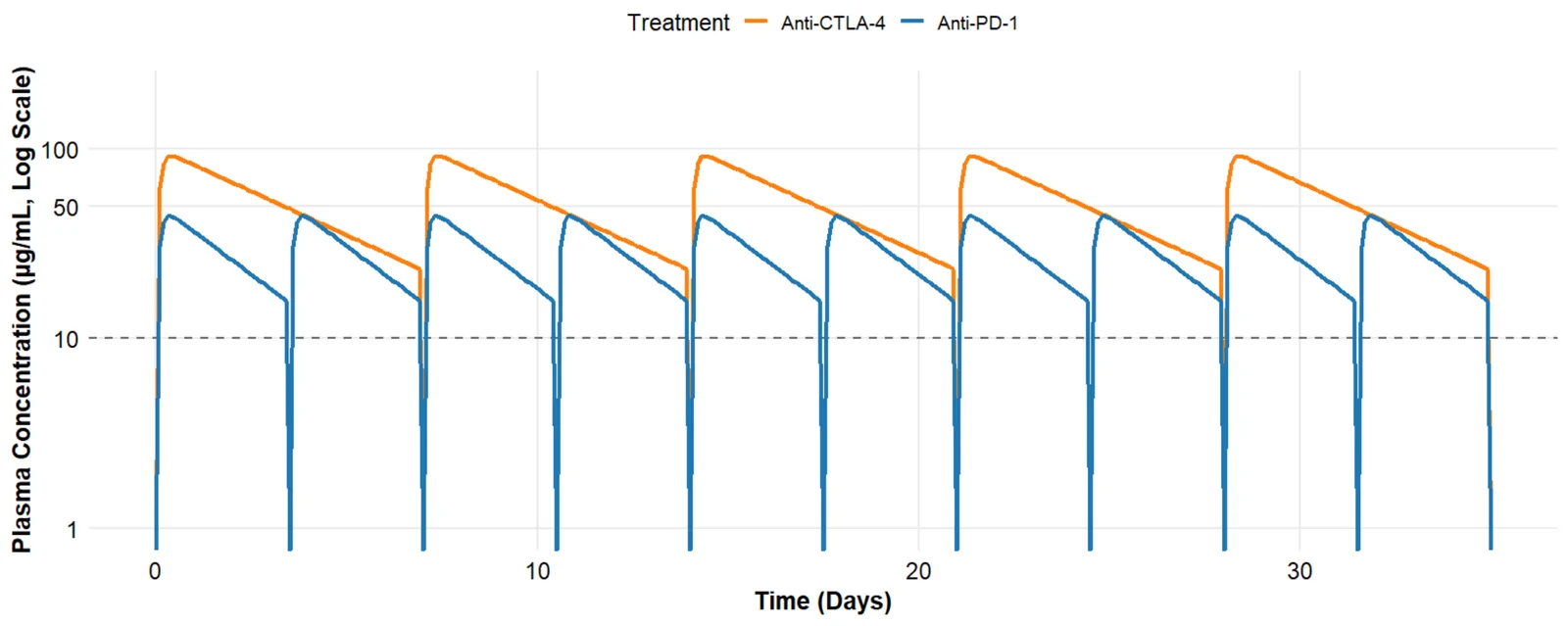
**Simulated intraperitoneal (IP) pharmacokinetic profiles of anti-PD-1 and anti-CTLA-4 in mice, presented on a semi-logarithmic scale.** This figure presents simulated data generated in silico and does not represent experimental data obtained in the present study. The aim of this simulation is to illustrate the pharmacokinetic behavior of both antibodies at the selected doses and dosing frequencies, providing a rationale for the chosen administration schedule. The solid lines represent the simulated plasma concentration profiles following IP administration of anti-PD-1 (5 mg/kg every 3.5 days, blue) and anti-CTLA-4 (10 mg/kg every 7 days, orange), based on their reported murine half-lives (~2 days for anti-PD-1, [40]; ~3.25 days for the anti-CTLA-4 surrogate 9H10, [43]) and the prolonged tumor retention of anti-CTLA-4 antibodies [39]. The horizontal dashed line indicates an approximate therapeutic threshold (~10 µg/mL) corresponding to the estimated 90% receptor saturation. While anti-CTLA-4 remains above this threshold throughout the 7-day interval, the rapid clearance of anti-PD-1 necessitates a more frequent dosing schedule to maintain adequate systemic exposure. Generated using R (version 4.6.1; R Foundation, Vienna, Austria) and RStudio (version 2026.07.1+147.pro6; Posit Software, Boston, MA, USA), with the ggplot2, dplyr, and tidyr packages.

**Figure 2:**
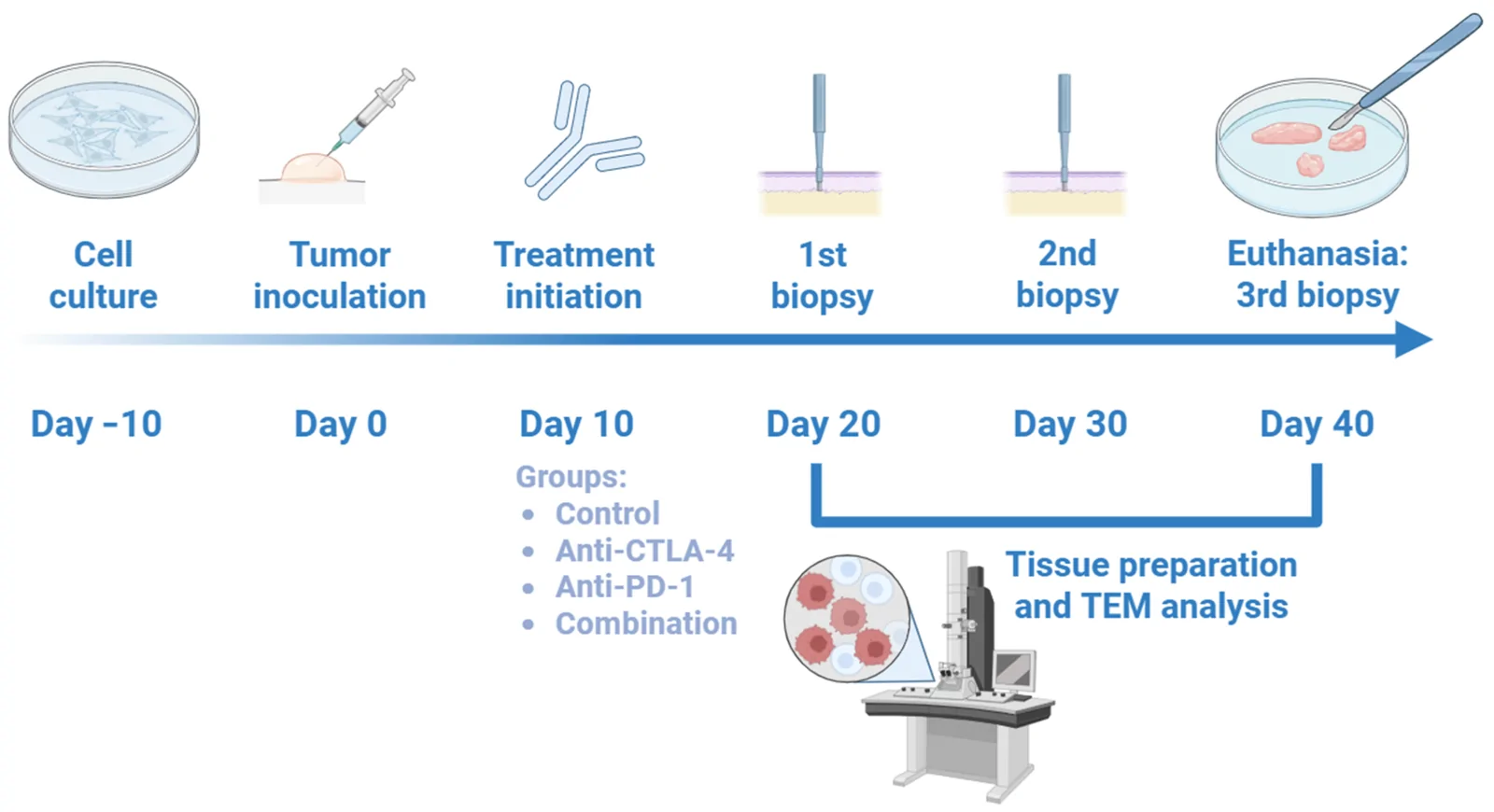
**Schematic overview of the experimental study design.** B16F10 melanoma-bearing C57BL/6 mice were randomized into four experimental groups (Control, Anti-CTLA-4, Anti-PD-1, and Combination) and treated from Day 10 to Day 40. Skin biopsies were collected on Days 20 and 30, and at the experimental endpoint (Day 40) following euthanasia. All tissue samples were processed for tissue preparation and transmission electron microscopy (TEM) analysis. Created in BioRender. Karakousis, V.A. (2026).

**Figure 3:**
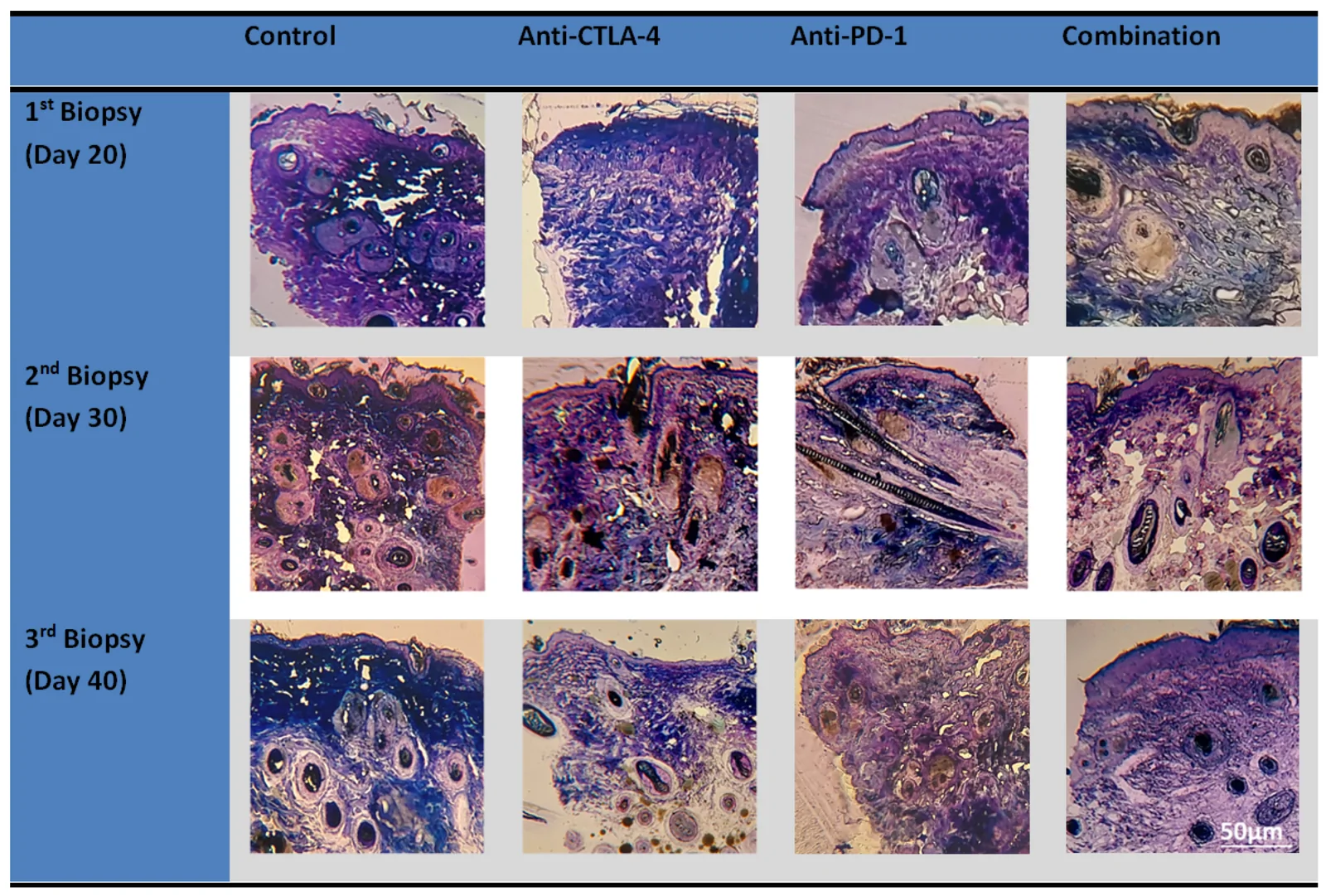
**Representative semi-thin sections stained with toluidine blue.** Columns represent the treatment groups (Control, Anti-CTLA-4, Anti-PD-1, and Combination), while rows represent the three biopsy time points (Days 20, 30, and 40). Magnification: ×10. Minor variations in staining intensity across sections are attributable to technical factors during the standard staining procedure (e.g., differential dye uptake) and do not reflect underlying biological differences between groups.

**Figure 4:**
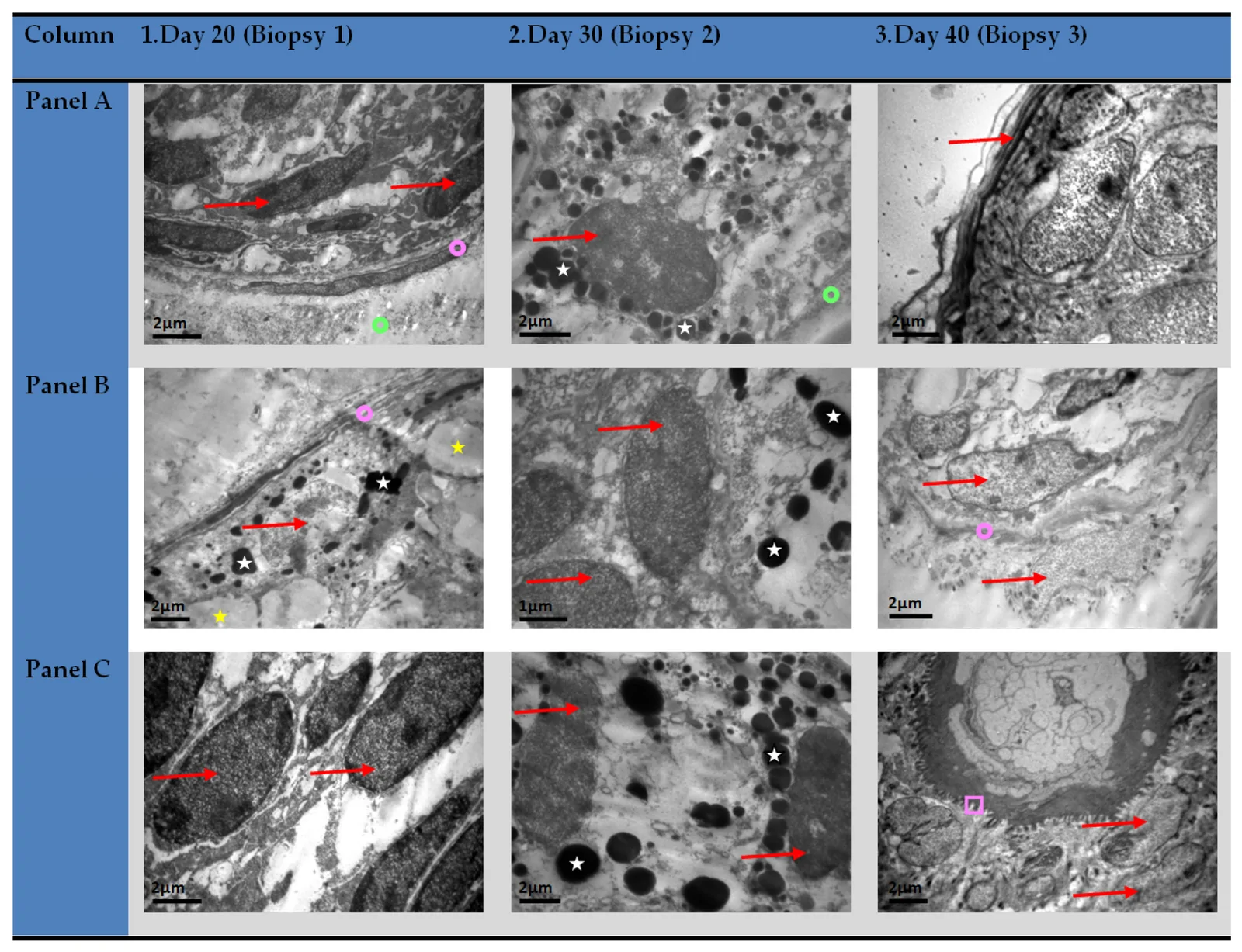
**Ultrastructural analysis of the tumor microenvironment in the Control group across treatment time points.** B16-F10 melanoma cells were identified based on characteristic ultrastructural features, including the presence of melanosomes at various developmental stages [63,64,65], irregular and tightly packed nuclei, and the presence of lipid droplets, which may serve as an energy source [64]. (**A1**) Tumor front at Day 20 showing ischemic densely packed B16 cells, pseudocapsule formation by flattened fibroblasts, healthy dermis with normally organized collagen, and the tumor front exhibiting degraded collagen and intercellular edema. (**B1**) Day 20 overview of the dermal–tumor interface showing keratinocytes, melanosomes, fibrocytes, and lipid droplets. (**C1**) Day 20 showing the stacking of B16-F10 melanoma cells with peripheral melanosomes. (**A2**) Viable B16 cell at Day 30 with cytoplasm rich in melanosomes, normal mitochondria, dilated ER, nuclear envelope, nucleolus, autolysosomes, and cytoplasmic membrane; melanosome polarization is evident on one side of the nucleus. (**B2**) Day 30 showing the stacking of B16-F10 melanoma cells with peripheral melanosomes. (**C2**) Day 30 displaying perinuclear clustering of melanosomes. (**A3**) Day 40 showing epidermal invasion by melanoma cells. (**B3**) Day 40 showing the tumor invasion front where B16 melanoma cells breach the fibrous capsule/barrier, providing an overview of the tumor-stroma contact zone; structural integrity of the fibrous barrier and its breach by cancer cell nuclei are consistent with the process of local invasion and ECM disruption. (**C3**) Day 40 showing a pathological tumor microvessel with an aberrant vascular wall, membrane projections in a close contact zone (perivascular niche), and B16 cell nuclei in a perivascular arrangement. Symbols: Red arrows indicate apoptotic or invasive tumor cells (B16); Yellow stars indicate lipid droplets; White stars indicate melanosomes; Green circles indicate normal extracellular matrix/stroma; Magenta circles indicate pathological fibrosis/fibrous capsule (ECM remodeling); Magenta squares indicate the aberrant vascular wall/perivascular niche.

**Figure 5:**
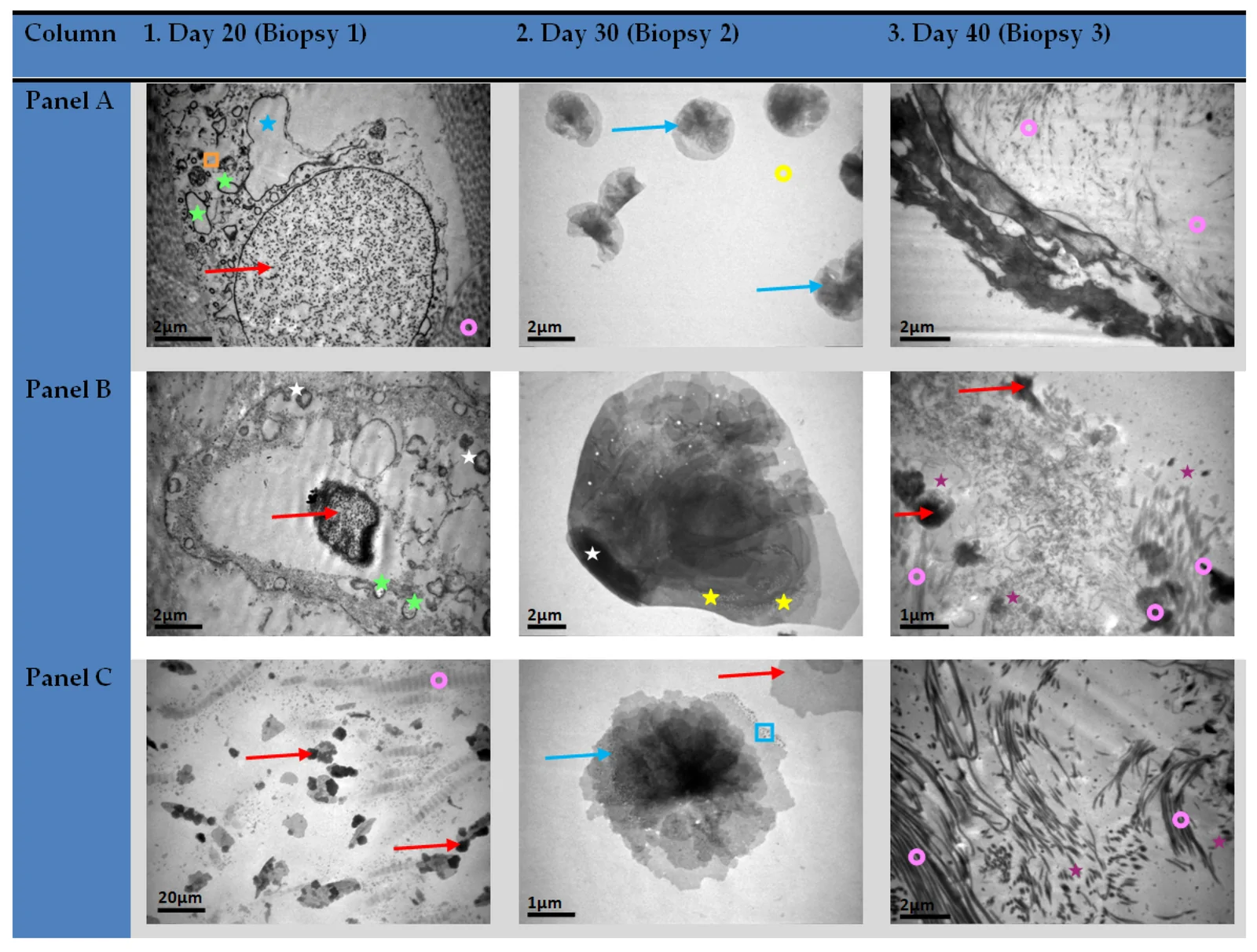
**Ultrastructural analysis of the tumor microenvironment in the Anti-CTLA-4 group across treatment time points.** The identification of immune cells (lymphocytes and macrophages) in this study is based exclusively on morphological criteria observed via transmission electron microscopy (TEM), such as nuclear shape, size, and specific cytoplasmic organelles. Definitive characterization would require complementary techniques, such as immunoelectron microscopy with specific markers (e.g., CD3 for T cells, CD68 or F4/80 for macrophages), which were outside the scope of this ultrastructural analysis. Lymphocytes were identified based on established ultrastructural criteria, including a high nuclear-to-cytoplasmic ratio, large and irregular nuclei, and the presence of characteristic cytoplasmic organelles [66], as well as distinct electron-dense lytic granules [67]. Given that these morphological features do not allow for the definitive discrimination between distinct lymphocyte subsets (e.g., CD4+ vs. CD8+ T cells), the identification in this study refers to lymphocytes in general. Macrophages were identified based on their characteristic ultrastructure, including an irregular nucleus and a cytoplasm rich in lipid droplets. Given the additional presence of melanin aggregates, these cells were characterized as foamy macrophage-like cells (melanophage-like cells), a phenotype consistent with the immunosuppressive and lipid-laden state observed in the tumor microenvironment [68]. In addition, the presence of cholesterol crystals within phagolysosomes was noted, consistent with lysosomal damage and inflammasome activation [69]. Accordingly, all references to lymphocytes in this study refer to lymphocytes in general, whereas the terms ‘foamy macrophage-like cells’ and ‘M2-like’ are used to denote morphological and phenotypic similarities, respectively, without definitive subset characterization. (**A1**) B16 melanoma cell at Day 20 showing ER cisternal dilation, swollen mitochondria, autolysosomes, and chromatinolysis; peritumoral fibrosis is evident. (**B1**) Apoptotic B16 cell at Day 20 with pyknotic nucleus, swollen mitochondria, degrading melanosomes, and cytoplasmic rarefaction. (**C1**) Day 20 overview showing apoptotic B16 cells, and collagen fibers with striations. (**A2**) Cluster of mature lymphocytes at Day 30 in acellular stroma (immune surveillance). (**B2**) Activated foamy macrophage-like cell (melanophage-like cell) at Day 30 containing a melanophagolysosome with melanin aggregates, extensive cytoplasmic vacuolization (foamy appearance), and lipid droplet sites. (**C2**) Detail of an activated lymphocyte at Day 30 with pseudopodia, a multilamellar body, and an adjacent B16 cell fragment. (**A3**) Day 40 showing post-treatment subcutaneous fibrosis and replacement of neoplastic tissue. (**B3**) Day 40 showing prominent pathological deposition of dense, wavy collagen fibril bundles coexisting with cellular debris, reflecting ECM remodeling and tissue response following therapy. (**C3**) Day 40 showing extensive pathological remodeling and ECM degradation within the fibrosis zone, featuring disorganized and fragmented collagen fibrils, cellular debris, and melanosome remnants, indicative of metabolic stress and tissue destruction. Symbols: Red arrows indicate apoptotic or invasive tumor cells (B16); Cyan arrows indicate infiltrating lymphocytes; Cyan squares indicate polarized lytic granules; Orange squares indicate autolysosomes; White stars indicate melanosomes; Green stars indicate swollen mitochondria; Blue stars indicate ER cisternal dilation; Yellow stars indicate lipid droplets; Dark purple stars indicate cellular/extracellular debris; Yellow circles indicate acellular stroma (tumor bed); Magenta circles indicate pathological fibrosis/fibrous capsule (ECM remodeling).

**Figure 6:**
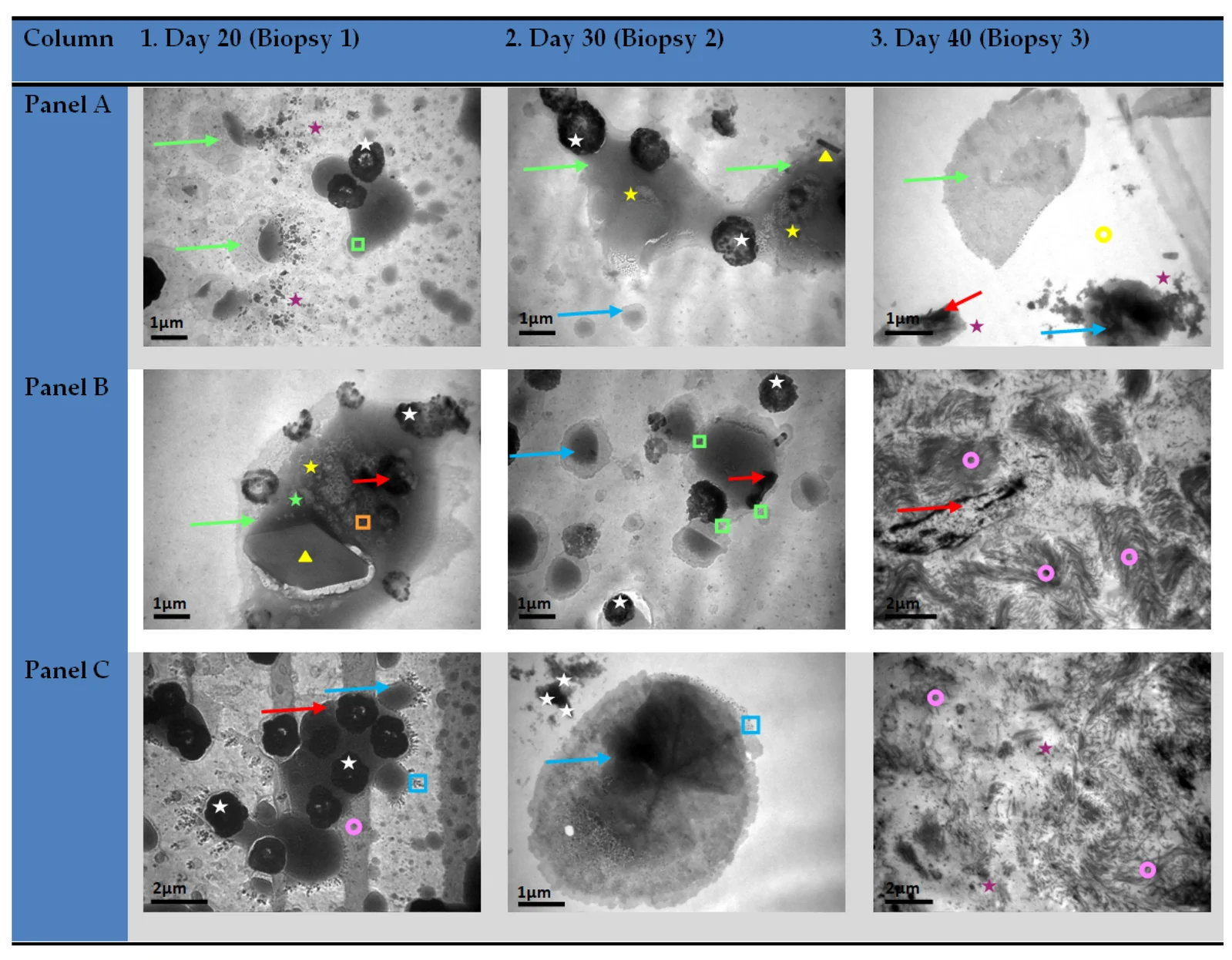
**Ultrastructural analysis of the tumor microenvironment in the Anti-PD-1 group across treatment time points.** (**A1**) Day 20 overview showing infiltrating lymphocytes, apoptotic bodies, immunological synapses, and phagocytic cups. (**B1**) Day 20 showing a foamy macrophage-like cell with a cholesterol crystal, autolysosome, lipid droplets, melanized apoptotic bodies, a condensed B16 nucleus, signs of metabolic overload, and lymphocyte interaction/immune cross-talk. (**C1**) Day 20 showing an apoptotic B16 cell, lymphocytes’ uropods, cytotoxic granules, and fibroblast pro-cesses. (**A2**) Day 30 showing a macrophage with ultrastructural features consistent with activation and phagocytosis of melanized apoptotic bodies, lymphocytes with morphological signs of activa-tion, features suggestive of fatty acid oxidation, lipid sequestration, characteristics consistent with metabolic reprogramming, and cytotoxicity (perforin/granzyme). (**B2**) Day 30 showing lymphocyte with features suggestive of clonal expansion/mitosis, nuclear pyknosis, chromatin condensation, efferocytosis, an immunological synapse, and lymphocyte–macrophage cross-talk. (**C2**) Day 30 showing a lymphocyte with polarized lytic granules, and melanosome remnants. (**A3**) Day 40 showing a macrophage with lysosomes and euchromatic nucleus, a lymphocyte interacting with cellular debris, and acellular matrix. (**B3**) Day 40 showing a dense fibrotic scar, pathological fibrosis, disorganized collagen bundles, and a trapped B16 cell nucleus within the fibrous stroma, suggesting the entrapment of neoplastic cells by the extensive ECM remodeling. (**C3**) Day 40 showing evidence of metabolic stress within the fibrosis zone, featuring disorganized collagen fibrils, cellular debris, and melanosome remnants. Symbols: Red arrows indicate apoptotic or invasive tumor cells (B16); Cyan arrows indicate infiltrating lymphocytes; Green arrows indicate macrophages with morphological features of activation/foaming; Cyan squares indicate polarized lytic granules; Green squares indicate immunological synapses; Orange squares indicate autolysosomes/phagolysosomes; White stars indicate melanosomes; Yellow stars indicate lipid droplets; Green stars indicate swollen mitochondria; Dark purple stars indicate extracellular debris/vesicular material; Magenta circles indicate pathological fibrosis/fibrous capsule (ECM remodeling); Yellow circles indicate acellular stroma (tumor bed); Yellow triangles indicate cholesterol crystals.

**Figure 7:**
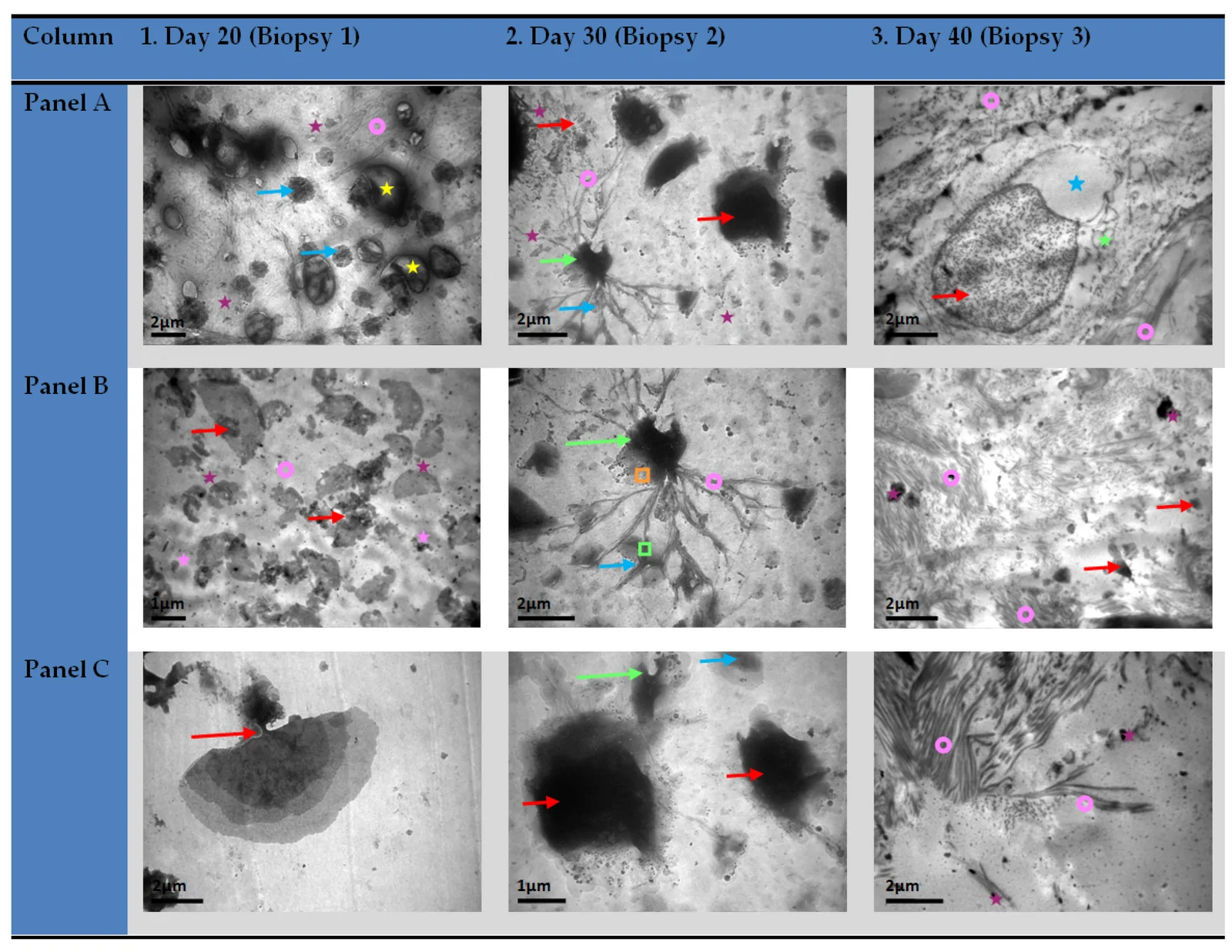
**Ultrastructural analysis of the tumor microenvironment in the Combination (An-ti-CTLA-4 + Anti-PD-1) group across treatment time points.** (**A1**) Day 20 overview showing in-filtrating lymphocytes, necrotic debris, necrotic blebs, lipid droplets, and disorganized collagen fibers. (**B1**) Day 20 showing a cytolytic zone with loss of stromal architecture, necrotic debris, and organelle ghosts. (**C1**) Day 20 showing rupture of an apoptotic body edge with release of dense granular aggregates/debris. (**A2**) Day 30 overview showing cellular debris, macrophages with cytoplasmic phagolysosomes, phagocytic cups, a collagen fiber network, and lymphocytes with polarized lytic granules. (**B2**) Day 30 showing a pioneer macrophage with a branching collagen fiber network and polarized infiltrating lymphocytes in intimate contact, and cytoplasmic phagolysosomes. (**C2**) Day 30 detail showing a phagocytic cup, infiltrating lymphocyte, phagocytes with B16 phagosomes and polarized vesicles, and collagen tracks. (**A3**) Day 40 showing a stressed B16 cell in the fibrosis zone with nuclear stress, dilated ER, swollen mitochondria, and dense, irregular collagen bundles. (**B3**) Day 40 showing fibrosis and biological debris, with melanosomes and B16 cell remnants incorporated within dense collagen bundles. (**C3**) Day 40 showing dense collagen fiber bundles, reflecting extensive fibrosis and extracellular matrix remodeling. Symbols: Red arrows indicate apoptotic or invasive tumor cells (B16); Green arrows indicate macrophages with morphological features of activation/foaming; Cyan arrows indicate infiltrating lymphocytes; Green squares indicate immunological synapses; Orange squares indicate autolysosomes/phagolysosomes; Yellow stars indicate lipid droplets; Green stars indicate swollen mitochondria; Magenta circles indicate pathological fibrosis/fibrous capsule (ECM remodeling); Blue stars indicate ER cisternal dilation; Magenta stars indicate organelle ghosts; Dark purple stars indicate extracellular de-bris/vesicular material.

**Figure 8:**
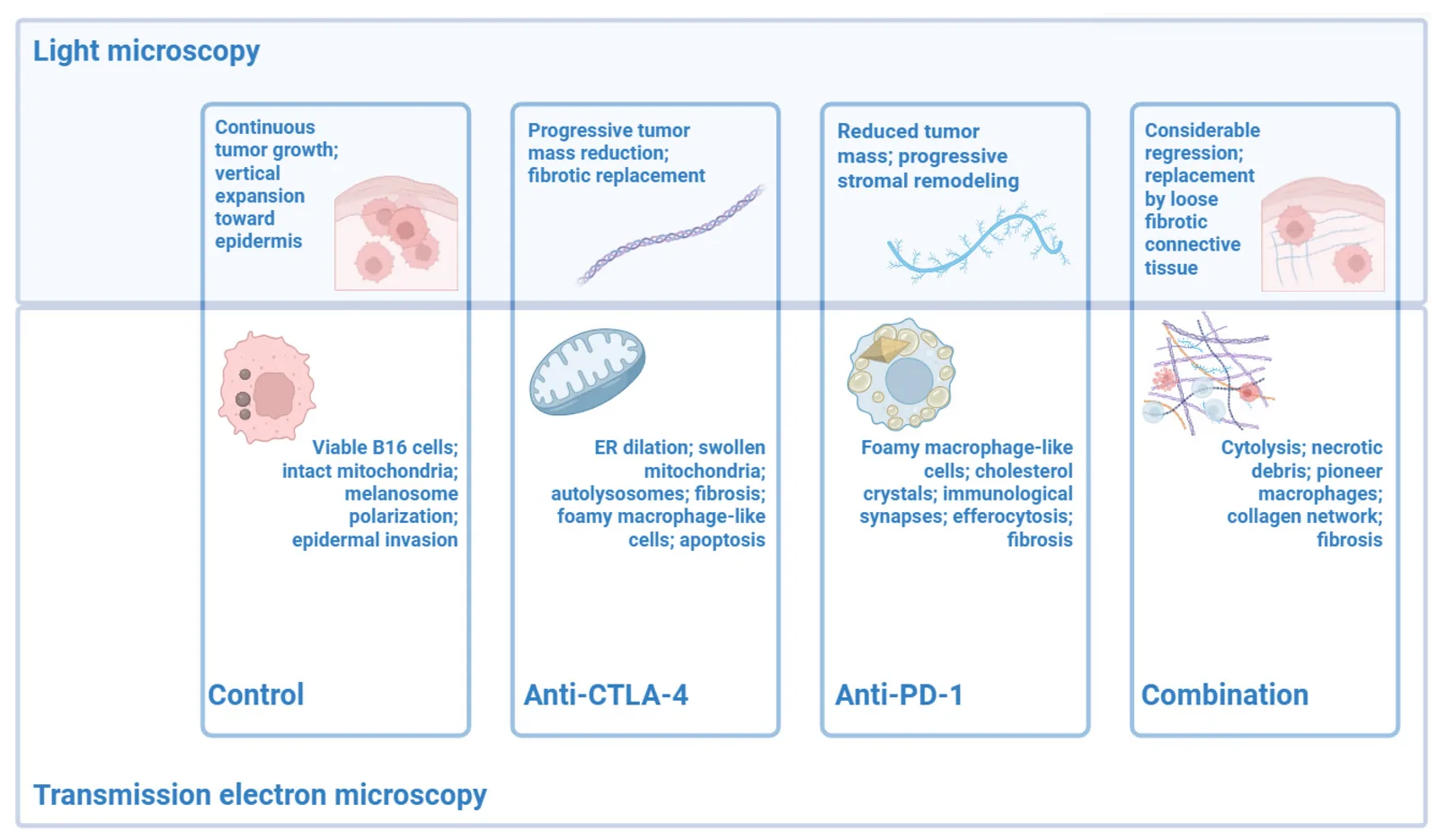
**Summary of the main light microscopic and ultrastructural findings across the four experimental groups.** The upper row summarizes the light microscopic observations (Figure 3), while the lower row summarizes the key ultrastructural features observed by transmission electron microscopy (Figure 4, Figure 5, Figure 6 and Figure 7). Control tumors exhibited continuous growth with viable B16 cells and intact mitochondria. Anti-CTLA-4 treatment was associated with ER dilation, mitochondrial swelling, apoptosis, and fibrosis. Anti-PD-1 treatment was characterized by foamy macrophage-like cells, cholesterol crystals, immunological synapses, and efferocytosis. Combination therapy was accompanied by extensive cytolysis, necrotic debris, pioneer macrophages, and dense collagen networks. Created in BioRender. Karakousis, V.A. (2026).

**Figure 9:**
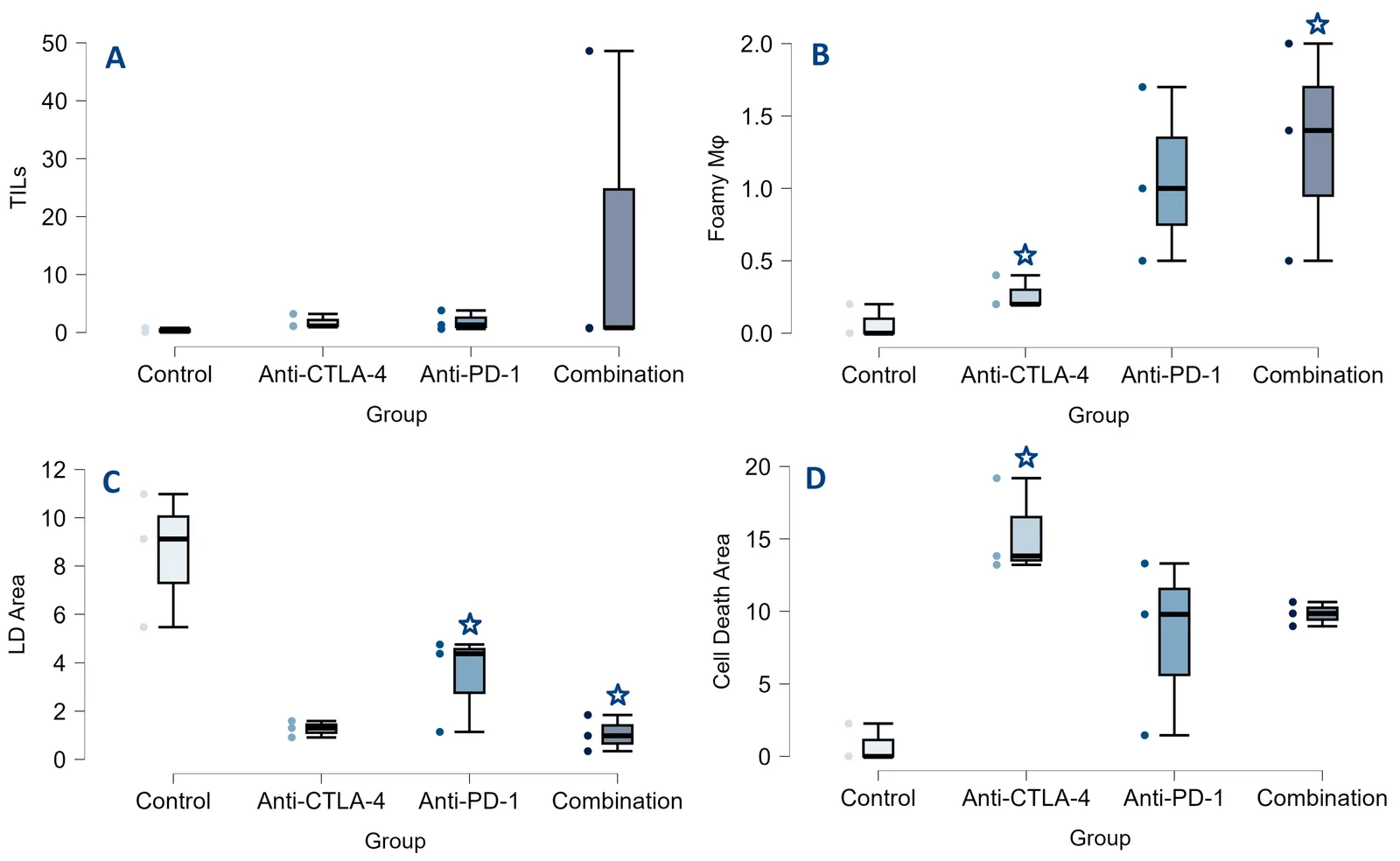
**Morphometric analysis of the peritumoral interface.** Box plots showing the distribution of (**A**) tumor-infiltrating lymphocytes (TILs), (**B**) foamy macrophage-like cells (Foamy Mφ), (**C**) lipid droplet area (LD Area), and (**D**) cell death area across the four experimental groups (Control, Anti-CTLA-4, Anti-PD-1, Combination). Individual mouse-level data points (*n* = 3 per group) are shown as overlaid dots. For clarity, data points are displayed alongside the box plots rather than overlaid on top of them, and no jitter was applied to preserve the exact position of each data point without altering the underlying values. Plots display the median (horizontal line within the box), the interquartile range (box), and the whiskers (min to max). Statistical significance was assessed using ANOVA (analysis of variance) or Kruskal–Wallis tests, followed by Dunn’s post hoc comparisons. Asterisks (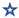) denote statistically significant differences compared to the Control group (*p* < 0.05).

**Figure 10:**
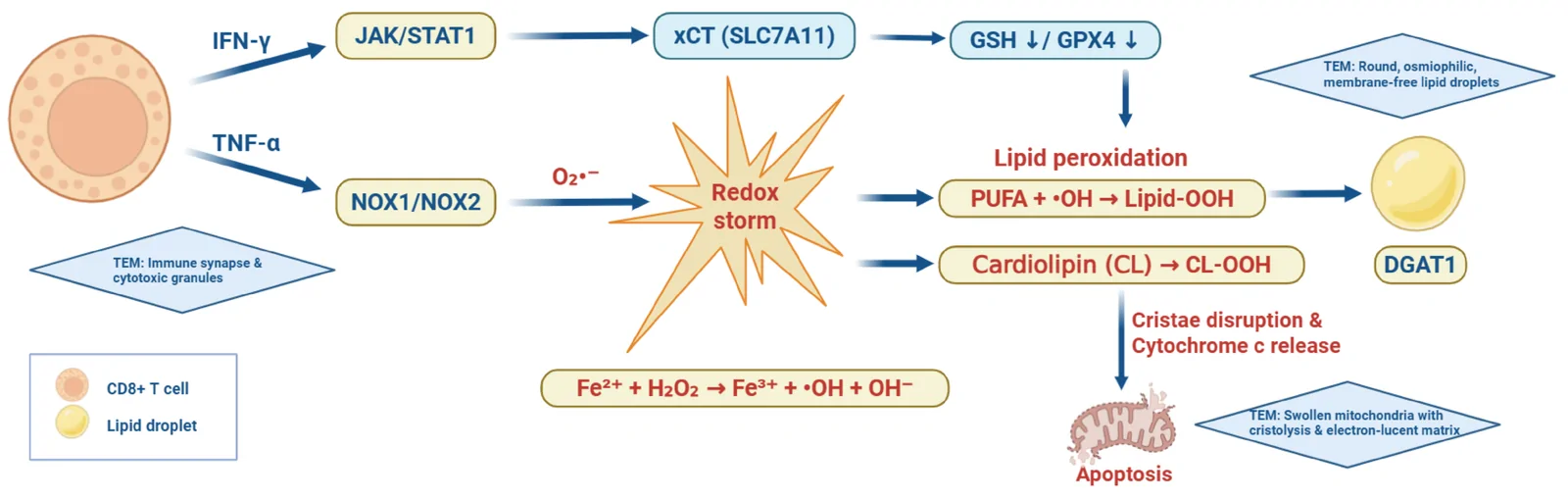
**Proposed pro-oxidant cascade, lipid peroxidation, and mitochondrial damage (Mechanisms 1–3).** The figure illustrates the established mechanism by which activated CD8^+^ T cells (based on literature) induce the redox storm through IFN-γ and TNF-α. TEM correlates from our analysis are shown in blue diamonds. The induction of the redox storm by activated lymphocytes and the consequent destruction of lipids and mitochondria are depicted. The cytokines IFN-γ and TNF-α activate the JAK/STAT1 and NOX1/NOX2 pathways, respectively, leading to GSH/GPX4 depletion and superoxide (O_2_•^−^) production. O_2_•^−^ fuels the Fenton reaction (Fe^2+^ + H_2_O_2_ → Fe^3+^ + •OH + OH^−^), generating the highly reactive hydroxyl radical (•OH). This radical drives lipid peroxidation (PUFA + •OH → Lipid-OOH), which promotes lipid droplet formation via DGAT1. Simultaneously, •OH triggers cardiolipin (CL) oxidation to CL-OOH, causing cristae disruption, cytochrome c release, and apoptosis. Morphological support is provided by TEM correlates for immune synapses, lipid droplets, and mitochondrial damage. Abbreviations: CL, cardiolipin; CL-OOH, oxidized cardiolipin; DGAT1, diacylglycerol O-acyltransferase 1; Fe^2+^/Fe^3+^, iron; GPX4, glutathione peroxidase 4; GSH, glutathione; H_2_O_2_, hydrogen peroxide; IFN-γ, interferon-γ; JAK/STAT1, Janus kinase/signal transducer and activator of transcription 1; Lipid-OOH, lipid hydroperoxide; NOX1/NOX2, NADPH oxidases 1 and 2; O_2_•^−^, superoxide radical; PUFA, polyunsaturated fatty acid; TEM, Transmission Electron Microscopy; TNF-α, tumor necrosis factor-α; xCT/SLC7A11, cystine/glutamate transporter; •OH, hydroxyl radical. Created in BioRender. Karakousis, V.A. (2026).

**Figure 11:**
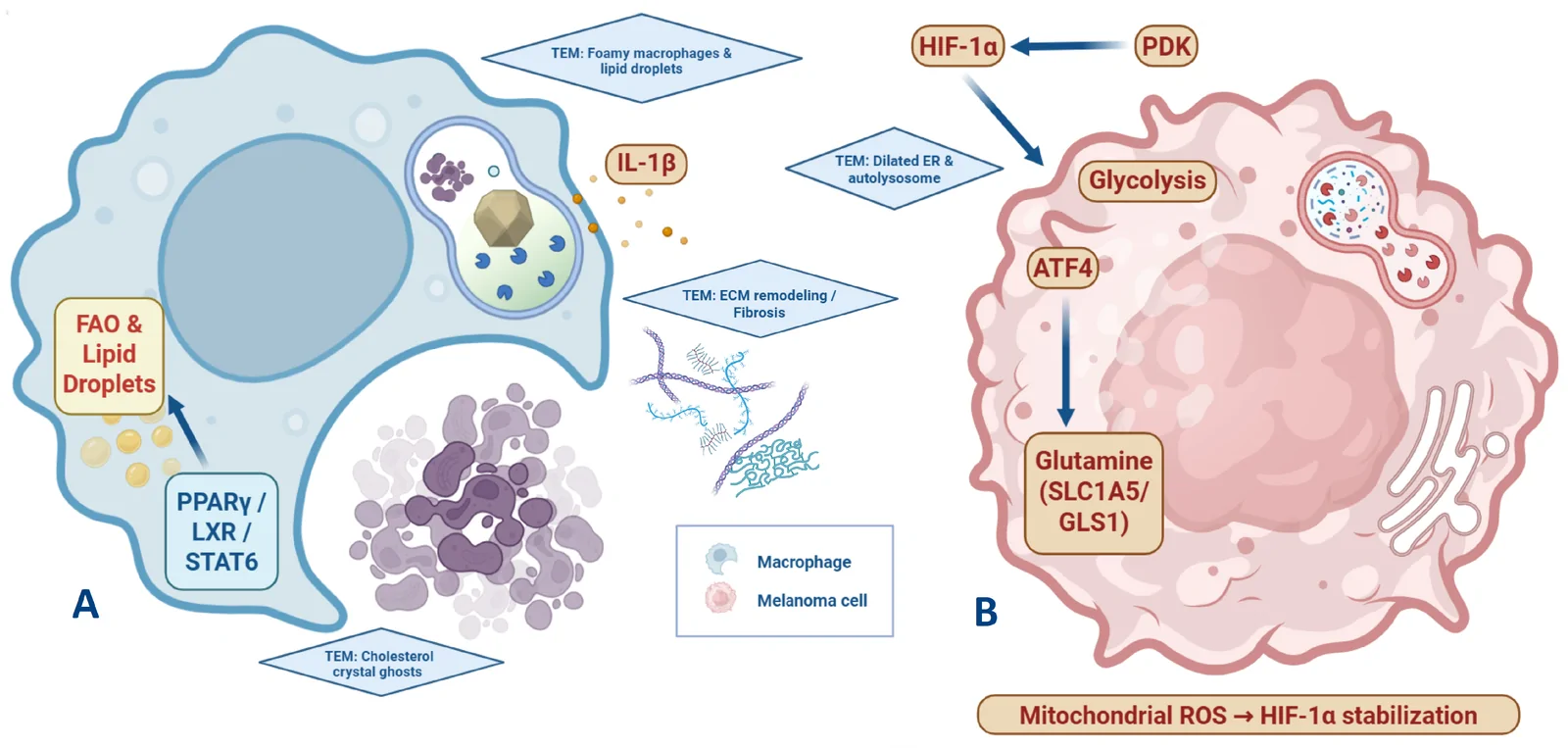
**Proposed immunosuppressive loop and metabolic plasticity (Mechanisms 4–6).** The figure illustrates the established mechanisms (based on literature) of the immunosuppressive loop and metabolic plasticity. TEM correlates from our analysis are shown in blue diamonds. The efferocytosis-driven immunosuppressive reprogramming of macrophages and the metabolic adaptation of surviving melanoma cells are depicted. (**A**) Macrophage efferocytosis of apoptotic tumor cells leads to cholesterol crystal formation within the phagolysosome, its disruption, and NLRP3 inflammasome activation with subsequent IL-1β release. Concurrently, PPARγ/LXR/STAT6 signaling drives fatty acid oxidation (FAO) and lipid droplet accumulation, generating foamy macrophage-like cells. The TEM correlate for ECM remodeling/fibrosis is consistent with the terminal fibrotic scar resulting from sustained M2-like activation. (**B**) Surviving melanoma cells engage metabolic plasticity. Mitochondrial ROS, generated from the initial oxidative assault, stabilize HIF-1α, which drives PDK-mediated glycolysis and metabolic lock. Simultaneously, ATF4 upregulates glutamine transporters (SLC1A5) and glutaminase (GLS1) to sustain TCA cycle anaplerosis, allowing cell survival. Morphological support is provided by TEM correlates for foamy macrophage-like cells, cholesterol crystal ghosts, dilated ER/autolysosomes, and ECM remodeling. Abbreviations: ATF4, activating transcription factor 4; ECM, extracellular matrix; ER, endoplasmic reticulum; FAO, fatty acid oxidation; GLS1, glutaminase 1; HIF-1α, hypoxia-inducible factor 1-alpha; IL-1β, interleukin-1β; LXR, liver X receptor; M2, alternatively activated macrophage; NLRP3, NLR family pyrin domain containing 3; PDK, pyruvate dehydrogenase kinase; PPARγ, peroxisome proliferator-activated receptor γ; ROS, reactive oxygen species; SLC1A5, glutamine transporter; STAT6, signal transducer and activator of transcription 6; TCA, tricarboxylic acid; TEM, Transmission Electron Microscopy. Created in BioRender. Karakousis, V.A. (2026).

**Figure 12:**
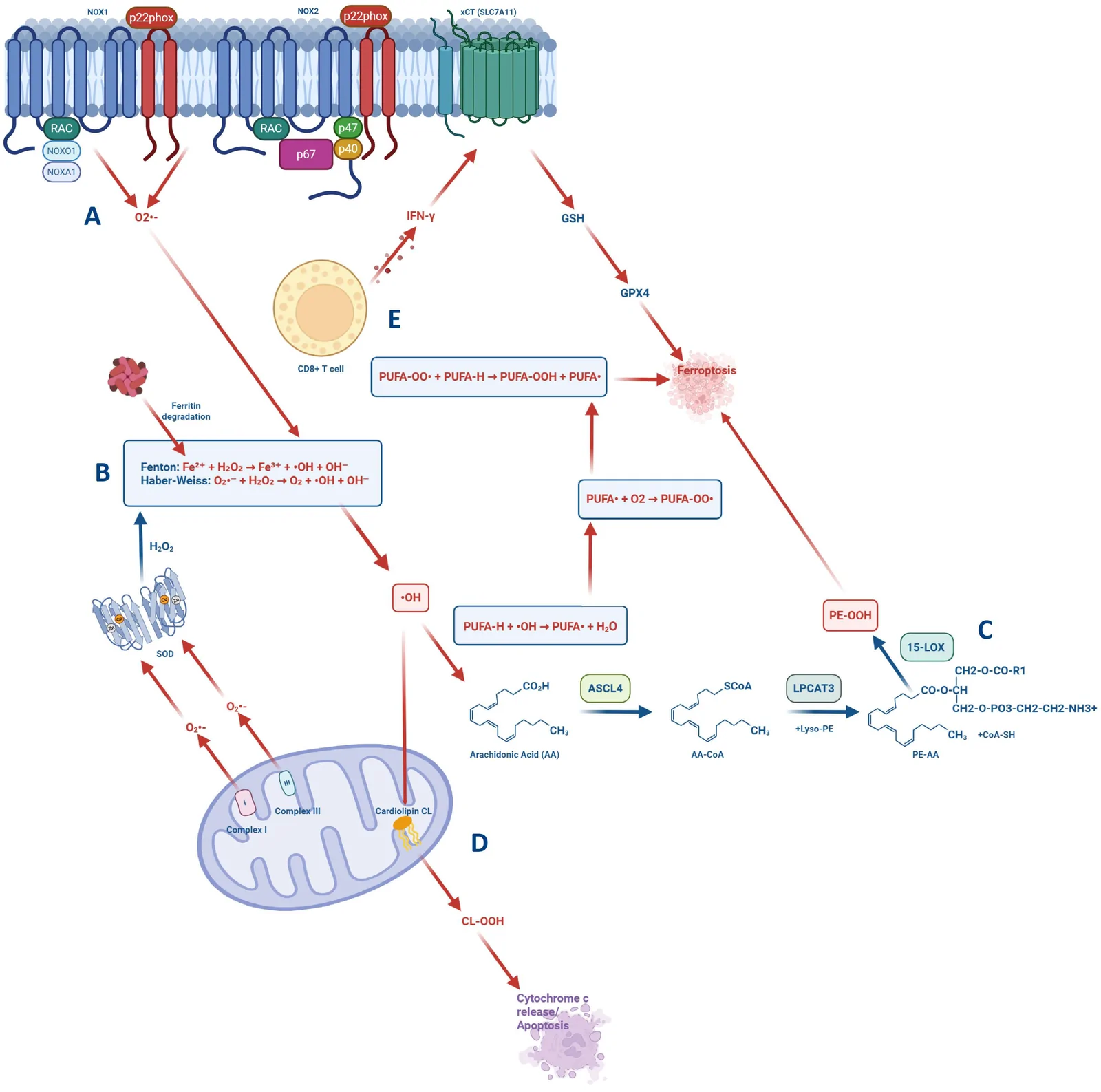
**Proposed chemical basis of the redox storm.** The figure illustrates the sequential generation and propagation of ROS, focusing on the enzymatic and non-enzymatic pathways of lipid peroxidation and mitochondrial damage, and the collapse of antioxidant defense. (A) NOX1/NOX2 and mitochondrial electron leakage from Complexes I/III generate superoxide (O_2_•^−^). Superoxide dismutase (SOD) converts O_2_•^−^ to H_2_O_2_. (B) Iron-driven Fenton chemistry (Fe^2+^ + H_2_O_2_ → Fe^3+^ + •OH + OH^−^) and the Haber–Weiss reaction (O_2_•^−^ + H_2_O_2_ →O_2_ + •OH + OH^−^) amplify the production of the highly toxic hydroxyl radical (•OH). (C) •OH initiates the peroxidation of membrane polyunsaturated fatty acids (PUFAs) via a self-propagating chain reaction (PUFA-H → PUFA• → PUFA-OO• → PUFA-OOH), while the enzymatic cascade involving ACSL4, LPCAT3, and 15-lipoxygenase generates the lethal phosphatidylethanolamine hydroperoxides (PE-OOH) that execute ferroptosis. (D) Simultaneously, •OH selectively oxidizes mitochondrial cardiolipin (CL) to CL-OOH, disrupting cristae architecture and triggering cytochrome c release and apoptosis. (E) The failure of the antioxidant shield, due to IFN-γ-mediated suppression of xCT, GSH depletion, and GPX4 inactivation, allows this redox storm to overwhelm the cell’s defenses, culminating in both ferroptotic and apoptotic cell death. Abbreviations: AA, arachidonic acid; ACSL4, acyl-CoA synthetase long-chain family member 4; CL, cardiolipin; CL-OOH, oxidized cardiolipin; CoA-SH, coenzyme A; Fe^2+^/Fe^3+^, iron; GSH, glutathione; GPX4, glutathione peroxidase 4; H_2_O_2_, hydrogen peroxide; IFN-γ, interferon-gamma; LPCAT3, lysophosphatidylcholine acyltransferase 3; Lyso-PE, lysophosphatidylethanolamine; NOXA1, NADPH oxidase activator 1; NOXO1, NADPH oxidase organizer 1; NOX1, NADPH oxidase 1; NOX2, NADPH oxidase 2; O_2_•^−^, superoxide anion; p22phox, p22 phagocyte oxidase; p40phox, p40 phagocyte oxidase; p47phox, p47 phagocyte oxidase; p67phox, p67 phagocyte oxidase; PE-AA, phosphatidylethanolamine-arachidonic acid; PE-OOH, oxidized phosphatidylethanolamine; PUFA, polyunsaturated fatty acid; RAC, Ras-related C3 botulinum toxin substrate; SLC7A11, solute carrier family 7 member 11; SOD, superoxide dismutase; xCT, cystine/glutamate antiporter; •OH, hydroxyl radical; 15-LOX, 15-lipoxygenase. Created in BioRender. Karakousis, V.A. (2026).

**Table 1:** **Summary of morphometric parameters across treatment groups (All data are presented as mean ± standard error of the mean** **(SEM)).**

Parameter	Control	Anti-CTLA-4	Anti-PD-1	Combination
**TILs (n/1000 µm^2^)**	0.37 ± 0.22	1.80 ± 0.70	1.90 ± 0.97	16.70 ± 15.95
**Foamy Mφ (n/1000 µm^2^)**	0.07 ± 0.07	0.27 ± 0.07	1.10 ± 0.35	1.30 ± 0.44
**LD Area (%)**	8.53 ± 1.62	1.27 ± 0.20	3.43 ± 1.15	1.06 ± 0.43
**Cell Death Area (%)**	0.76 ± 0.76	15.41 ± 1.90	8.18 ± 3.51	9.83 ± 0.48

## Data Availability

The light microscopy images of the peritumoral zone and the ultrastructural (TEM) images supporting the conclusions of this article are included in the article/Supplementary Material. The histological datasets (H&E, Masson’s trichrome, and immunohistochemistry for CD8 and α-SMA) and the plasma biochemical datasets are not publicly available yet, as they are part of separate manuscripts currently in preparation. These datasets are available from the corresponding author upon reasonable request.

## Supplementary Materials

The following supporting information can be downloaded at https://www.mdpi.com/article/10.3390/cells15191760/s1, Supplementary File S1: Complete ARRIVE 2.0 checklist and documentation of the protocol’s licensing; Supplementary File S2: Light microscopy images of the peritumoral zone; Supplementary File S3: TEM images across all treatment groups; Supplementary Tables; Table S1: Tumor volumes (mm^3^) in C57BL/6 mice bearing subcutaneous B16F10 melanoma across treatment groups; Table S2: Raw morphometric dataset of tumor-infiltrating lymphocytes (TILs), lipid droplet area (LD Area), foamy macrophages (Foamy Mφ), and cell death area (Cell Death Area) across treatment groups; Table S3: Descriptive statistics (mean, standard deviation, minimum, and maximum values) for all morphometric parameters; Table S4: Complete statistical outputs including ANOVA, Kruskal–Wallis, and Dunn’s post hoc comparisons.

## Abbreviations

The following abbreviations are used in this manuscript:

α-SMA	Alpha-Smooth Muscle Actin
ABCDE	Asymmetry, Border irregularity, Color variegation, Diam. > 6 mm, Evolution
ABCA1	ATP-binding cassette transporter A1
ABCG1	ATP-binding cassette transporter G1
AA	Arachidonic acid
ACSL4	Acyl-CoA Synthetase Long-Chain Family Member 4
AhR	Aryl hydrocarbon receptor
AJCC	American Joint Committee on Cancer
ALKAL1	ALK and LTK ligand 1
AMPK	AMP-activated Protein Kinase
ANOVA	Analysis of Variance
ARG1	Arginase 1
ASCT2	Alanine/Serine/Cysteine Transporter 2
ATF3	Activating Transcription Factor 3
ATF4	Activating Transcription Factor 4
ATG7	Autophagy-related gene 7
ATP	Adenosine Triphosphate
ATP11A/C	ATPase phospholipid transporting 11A/C
AUC	Area Under the Concentration–Time Curve
Axl	Axl receptor tyrosine kinase
BNIP3	BCL2 interacting protein 3
BRAF	v-Raf murine sarcoma viral oncogene homolog B
CCD	Charge-Coupled Device
CD4^+^	Cluster of differentiation 4 positive
CD8	Cluster of differentiation 8
CD8^+^	Cluster of differentiation 8 positive
CD28	Cluster of differentiation 28
CD36	Cluster of Differentiation 36
CD63	Cluster of Differentiation 63
CD80/CD86	Cluster of differentiation 80/86
CDKN2A	Cyclin-dependent kinase inhibitor 2A
CL	Cardiolipin
CL-OOH	Oxidized cardiolipin
CoA-SH	Coenzyme A
COX-2	Cyclooxygenase-2
CPT1	Carnitine Palmitoyltransferase 1
CSID	Chronically Sun-Damaged
CTLA-4	Cytotoxic T-lymphocyte-associated protein 4
DAMPs	Damage-Associated Molecular Patterns
DCA	Dichloroacetate
DGAT1	Diacylglycerol O-Acyltransferase 1
DPP4	Dipeptidyl Peptidase 4
DRP1	Dynamin-related protein 1
ECM	Extracellular matrix
EDTA	Ethylenediaminetetraacetic acid
EP2/EP4	E prostanoid receptors 2 and 4
ER	Endoplasmic reticulum
FAO	Fatty Acid Oxidation
FcγR	Fc gamma receptor
FcRn	Neonatal Fc receptor
Gas6	Growth arrest-specific 6
GCLC	Glutamate–cysteine ligase catalytic subunit
GLS1	Glutaminase 1
GLUT1	Glucose transporter 1
GNA11	G Protein Subunit Alpha 11
GNAQ	G Protein Subunit Alpha Q
GPX3	Glutathione peroxidase 3
GPX4	Glutathione Peroxidase 4
GSH	Glutathione
HG-T	Mitochondrial haplogroup T
HIF-1α	Hypoxia-Inducible Factor 1-alpha
HK2	Hexokinase 2
HMGB1	High Mobility Group Box 1
HMB45	Human Melanoma Black 45
HpETE	Hydroperoxyeicosatetraenoic acid
ICB	Immune Checkpoint Blockade
IDO	Indoleamine 2,3-dioxygenase
IFN-γ	Interferon-gamma
IgG	Immunoglobulin G
IgG1	Immunoglobulin G1
IL-1β	Interleukin-1 beta
IL-4	Interleukin-4
IL-10	Interleukin-10
IL-13	Interleukin-13
IL-24	Interleukin 24
immuno-EM	Immunoelectron microscopy
i.p.	Intraperitoneal
IRF1	Interferon Regulatory Factor 1
IRF4	Interferon Regulatory Factor 4
ISR	Integrated Stress Response
JAK	Janus Kinase
KIT	KIT Proto-Oncogene Receptor Tyrosine Kinase
Lamtor1	Late Endosomal/Lysosomal Adaptor and MAPK and mTOR Activator 1
LAP	LC3-associated phagocytosis
LC3	Microtubule-associated protein 1A/1B-light chain 3
LD	Lipid Droplet
LDH	Lactate Dehydrogenase
LDHA	Lactate dehydrogenase A
LPCAT3	Lysophosphatidylcholine Acyltransferase 3
LXR	Liver X receptor
LXRα	Liver X receptor alpha
Lyso-PE	Lysophosphatidylethanolamine
M1	Classically activated macrophage/Pro-inflammatory macrophage
M2	Alternatively activated macrophage
MAPK	Mitogen-activated protein kinase
MCGs	Multi-Core Granules
MCL1	MCL1 apoptosis regulator
Melan-A/MART-1	Melanoma antigen recognized by T cells 1
MerTK	Myeloid-epithelial-reproductive tyrosine kinase
MHC-I	Major Histocompatibility Complex class I
mtDNA	Mitochondrial DNA
MITF	Melanocyte-inducing transcription factor
mPGES-1	Microsomal prostaglandin E synthase-1
mregDC	Mature dendritic cell enriched in immunoregulatory molecules
MTOC	Microtubule-Organizing Center
mTORC1	Mechanistic Target of Rapamycin Complex 1
mTORC2	Mechanistic Target of Rapamycin Complex 2
Mφ	Macrophage
NADPH	Nicotinamide Adenine Dinucleotide Phosphate
NF1	Neurofibromin 1
NIX	NIP3-like protein X
NLRP3	NLR Family Pyrin Domain Containing 3
non-CSID	Non-Chronically Sun-Damaged
NOS2	Nitric Oxide Synthase 2
NOX	NADPH Oxidase
NOX1	NADPH Oxidase 1
NOX2	NADPH Oxidase 2
NOXA1	NADPH oxidase activator 1
NOXO1	NADPH oxidase organizer 1
NRAS	Neuroblastoma RAS viral oncogene homolog
NRF2	Nuclear Factor Erythroid 2-Related Factor 2
OXPHOS	Oxidative Phosphorylation
P38	p38 Mitogen-Activated Protein Kinase
p22phox	p22 phagocyte oxidase
p40phox	p40 phagocyte oxidase
p47phox	p47 phagocyte oxidase
p67phox	p67 phagocyte oxidase
PD	Pharmacodynamic
PD-1	Programmed cell death protein 1
PD-L1/PD-L2	Programmed cell death ligand 1/2
PDH	Pyruvate dehydrogenase
PDK	Pyruvate dehydrogenase kinase
PDK1	Pyruvate dehydrogenase kinase 1
PDK3	Pyruvate dehydrogenase kinase 3
PE-AA	Phosphatidylethanolamine-arachidonic acid
PE-OOH	Oxidized phosphatidylethanolamine
PGC-1α	Peroxisome proliferator-activated receptor gamma coactivator 1-alpha
PGE_2_	Prostaglandin E2
PHDs	Prolyl hydroxylases
PK	Pharmacokinetic
PPARγ	Peroxisome Proliferator-Activated Receptor Gamma
PRAME	PReferentially Expressed Antigen in Melanoma
PS	Phosphatidylserine
PTEN	Phosphatase and tensin homolog
PUFA	Polyunsaturated Fatty Acid
RAC	Ras-related C3 botulinum toxin substrate
ROS	Reactive Oxygen Species
S100	S100 calcium-binding protein
SOD	Superoxide dismutase
15-LOX	15-lipoxygenase
SAPE-OOH	1-stearoyl-2-15-HpETE-sn-glycero-3-PE
SCGs	Single-Core Granules
SEM	Standard Error of the Mean
SHP-2	Src homology 2 domain-containing phosphatase 2
SIRT3	Sirtuin 3
SLC1A5	Solute Carrier Family 1 Member 5
SLC3A2	Solute Carrier Family 3 Member 2
SLC7A11	Solute Carrier Family 7 Member 11
SmPC	Summary of Product Characteristics
SOX10	SRY-related HMG-box 10
STAT1	Signal Transducer and Activator of Transcription 1
STAT3	Signal Transducer and Activator of Transcription 3
STAT6	Signal Transducer and Activator of Transcription 6
TAM	Tyro3, Axl, MerTK receptor family
TAMs	Tumor-Associated Macrophages
TCA	Tricarboxylic acid cycle
TEM	Transmission Electron Microscopy
TGF-β	Transforming Growth Factor-beta
TILs	Tumor-Infiltrating Lymphocytes
TIM-4	T-cell immunoglobulin and mucin domain-containing molecule 4
TLR2	Toll-like receptor 2
TME	Tumor Microenvironment
TRAP1	TNF receptor-associated protein 1
T-sEVs	T-cell-derived Small Extracellular Vesicles
TNF-α	Tumor Necrosis Factor-alpha
TP53	Tumor protein p53
Treg	regulatory T cell
Tyro3	Tyrosine-protein kinase receptor TYRO3
UV	Ultraviolet
xCT	Cystine/Glutamate Antiporter
XKR8	XK-related protein 8
