# Supplementary material for: Redox Storm in the Tumor Microenvironment (ΤΜΕ): Multifaceted Redox Injury and Immunometabolic Reprogramming Induced by Immune Checkpoint Blockade in B16F10 Melanoma: An Ultrastructural Study of the Peritumoral Interface
Source: Cells. 2026 Sep 27;15(19):1760. doi: 10.3390/cells15191760 (PMC13664297; doi:10.3390/cells15191760)
Supplement: Supplementary file 1 [file cells-15-01760-s001.zip › cells-4584146-supplementary.pdf]

Supplementary Table S1. Tumor volumes (mm³) in C57BL/6 mice bearing subcutaneous B16F10 melanoma; mice were treated with Control, Anti-CTLA-4, Anti-PD-1, or Combination. Tumor volume was measured with a digital caliper and calculated using $V = (L \times W^2) / 2$. Individual tumor volumes for three mice per group are shown for Days 0, 20, 30, and 40. Data are also presented as mean ± standard deviation (SD).

Group	Mouse ID	Day 0 (mm^3)	Day 20 (mm^3)	Day 30 (mm^3)	Day 40 (mm^3)
Control	C1	0	122	1,118	2,498
	C2	0	153	1,008	2,002
	C3	0	116	919	1,479
	Mean ± SD	0.00 ± 0.00	130.3 ± 19.9	1015.0 ± 99.7	1993.0 ± 509.6
Anti-CTLA-4	I1	0	107	734	1,590
	I2	0	95	778	1,657
	I3	0	92	650	1,254
	Mean ± SD	0.00 ± 0.00	98.0 ± 7.9	720.7 ± 65.0	1500.3 ± 215.9
Anti-PD-1	N1	0	117	893	1,916
	N2	0	99	851	1,705
	N3	0	103	948	1,937
	Mean ± SD	0.00 ± 0.00	106.3 ± 9.5	897.3 ± 48.6	1852.7 ± 128.3
Combination	IN1	0	49	569	1,106
	IN2	0	78	502	877
	IN3	0	63	648	1,369
	Mean ± SD	0.00 ± 0.00	63.3 ± 14.5	573.0 ± 73.1	1117.3 ± 246.2

Supplementary Table S2. Individual FOV measurements of Tumor-Infiltrating Lymphocytes (TILs), Foamy Macrophages (Foamy Mϕ), Lipid Droplet (LD) Area, and Cell Death Area across Control, Anti-CTLA-4, Anti-PD-1, and Combination groups.

Mouse ID	Group	FOV	TILs (n/1000 μm^2)	Foamy Mϕ (n/1000 μm^2)	LD Area (%)	Cell Death Area (%)
C1	**Control**	1	0	0	23.33	0

		2	0	0	11.65	0
		3	0	0	1.71	0
		4	0	0	1.22	0
		5	0	0	1.30	0
		6	0	0	10.92	0
		7	0	0	38.74	0
		8	0	0	0.40	0
		9	2	0	0.24	0
		10	0	0	1.65	0
**C2**	**Control**	1	1	0	0.00	6.06
		2	0	2	0.09	0
		3	0	0	0.00	16.61
		4	0	0	23.09	0
		5	0	0	26.81	0
		6	0	0	23.63	0
		7	0	0	4.66	0
		8	0	0	8.75	0
		9	0	0	22.80	0
		10	0	0	0.00	0
**C3**	**Control**	1	8	0	0.00	0
		2	0	0	11.16	0
		3	0	0	7.79	0
		4	0	0	9.39	0
		5	0	0	4.66	0
		6	0	0	8.71	0
		7	0	0	7.90	0
		8	0	0	0.54	0

		9	0	0	4.17	0
		10	0	0	0.46	0
**I1**	**Anti-CTLA-4**	1	0	0	0.00	12.33
		2	0	0	0.00	3.42
		3	0	0	7.51	0
		4	0	0	1.60	0
		5	0	0	0.00	22.36
		6	3	0	0.00	6.03
		7	2	0	0.00	29.81
		8	0	0	0.00	28.82
		9	3	0	0.00	23.27
		10	3	2	0.00	12.14
**I2**	**Anti-CTLA-4**	1	0	2	0.62	1.83
		2	14	0	0.00	28.70
		3	5	0	0.00	15.67
		4	0	1	13.29	3.50
		5	0	0	0.00	6.48
		6	1	1	1.97	6.78
		7	4	0	0.00	43.88
		8	0	0	0.00	42.32
		9	8	0	0.00	35.25
		10	0	0	0.00	7.49
**I3**	**Anti-CTLA-4**	1	1	1	0.60	2.93
		2	2	0	0.00	23.51
		3	0	0	0.00	3.48
		4	2	0	0.00	5.72
		5	1	0	0.00	2.98

		6	0	0	5.62	0
		7	2	0	0.00	0
		8	0	1	6.77	28.51
		9	2	0	0.00	17.72
		10	1	0	0.00	47.20
**N1**	**Anti-PD-1**	1	2	4	0.72	7.57
		2	1	2	2.98	7.36
		3	0	1	17.26	0
		4	0	2	2.39	20.61
		5	2	3	6.02	16.55
		6	0	2	4.52	20.36
		7	0	0	0.00	25.40
		8	0	1	4.49	0
		9	1	1	2.99	0
		10	0	1	2.44	0
**N2**	**Anti-PD-1**	1	2	1	3.50	0
		2	6	0	0.00	0
		3	4	2	0.81	0
		4	1	2	3.70	0
		5	0	0	10.37	0
		6	0	0	10.05	0
		7	0	0	10.05	0
		8	0	0	0.00	14.56
		9	0	0	6.91	0
		10	0	0	2.24	0
**N3**	**Anti-PD-1**	1	0	0	2.00	0
		2	0	0	2.13	0

		3	3	0	0.00	30.43
		4	10	3	0.82	9.44
		5	12	2	1.49	18.84
		6	0	0	0.00	25.88
		7	0	0	0.00	37.89
		8	1	2	1.26	10.54
		9	2	3	3.71	0
		10	10	0	0.00	0
**IN1**	**Combination**	1	0	0	0.00	7.15
		2	3	0	0.00	11.53
		3	2	1	0.74	3.05
		4	0	2	0.51	0.72
		5	1	1	1.30	1.32
		6	0	1	0.88	6.46
		7	0	0	0.00	6.46
		8	0	0	0.00	27.15
		9	0	0	0.00	17.68
		10	2	0	0.00	8.41
**IN2**	**Combination**	1	0	0	0.00	7.47
		2	2	2	4.08	9.27
		3	0	4	6.33	0
		4	0	0	0.00	14.20
		5	1	3	1.08	13.77
		6	2	0	0.00	23.63
		7	0	3	2.84	9.63
		8	0	0	0.00	6.78
		9	2	2	4.11	3.43

IN3	Combination	10	0	0	0.00	10.30
		1	0	0	0.00	13.76
		2	8	5	1.57	1.86
		3	18	6	0.77	5.61
		4	30	2	0.83	25.56
		5	24	2	0.78	8.53
		6	12	2	0.34	22.13
		7	8	2	5.04	10.10
		8	184	0	0.00	0
		9	171	3	0.51	2.51
		10	31	0	0.00	16.30

Supplementary Table S3. Descriptive statistics (mean, standard deviation, minimum, and maximum) for all morphometric parameters (TILs, Foamy Mϕ, LD Area, and Cell Death Area) across the four treatment groups.

Descriptive Statistics							
Parameter	Group	Valid	Missing	Mean	Std. Deviat.	Minimum	Maximum
TILs	**Control**	3	0	0.367	0.379	0.100	0.800
	**Anti-CTLA-4**	3	0	1.800	1.212	1.100	3.200
	**Anti-PD-1**	3	0	1.900	1.682	0.600	3.800
	**Combination**	3	0	16.70	27.63	0.700	48.60
Foamy Mϕ	**Control**	3	0	0.067	0.115	0.000	0.200
	**Anti-CTLA-4**	3	0	0.267	0.115	0.200	0.400
	**Anti-PD-1**	3	0	1.067	0.603	0.500	1.700
	**Combination**	3	0	1.300	0.755	0.500	2.000
LD Area	**Control**	3	0	8.527	2.798	5.480	10.98
	**Anti-CTLA-4**	3	0	1.267	0.341	0.910	1.590

	**Anti-PD-1**	3	0	3.427	1.989	1.140	4.760
	**Combination**	3	0	1.053	0.753	0.340	1.840
**Cell Death Area**	**Control**	3	0	0.757	1.311	0.000	2.270
	**Anti-CTLA-4**	3	0	15.41	3.291	13.21	19.19
	**Anti-PD-1**	3	0	8.183	6.081	1.460	13.30
	**Combination**	3	0	9.827	0.825	8.990	10.64

Supplementary Table S4. Detailed statistical outputs (ANOVA, Kruskal-Wallis, and Dunn's post hoc comparisons) for TILs, Foamy Mϕ, LD Area, and Cell Death Area.

A. TILs

ANOVA - TILs

Cases	Sum of Squares	df	Mean Square	F	p
**Group**	534.2	3	178.1	0.928	.470
**Residuals**	1535	8	191.9		

Note. **Type III Sum of Squares**

Kruskal-Wallis Test

					95% CI for Rank ε^2	
**Factor**	Statistic	df	p	Rank ε^2	Lower	Upper
**Group**	4.325	3	.228	0.393	0.242	0.917

Dunn's Post Hoc Comparisons - Group

Comparison	z	W_i	W_j	r_{rb}	p	p_{Bonf}	p_{Holm}
**Control - Anti-CTLA-4**	-1.875	2.833	8.333	1.000	.061	.365	.365
**Control - Anti-PD-1**	-1.648	2.833	7.667	0.778	.099	.597	.497
**Control - Combination**	-1.477	2.833	7.167	0.667	.140	.838	.559

Anti-CTLA-4 - Anti-PD-1	0.227	8.333	7.667	0.111	.820	1.000	1.000
Anti-CTLA-4 - Combination	0.398	8.333	7.167	-0.333	.691	1.000	1.000
Anti-PD-1 - Combination	0.170	7.667	7.167	0.111	.865	1.000	1.000

Note. Rank-biserial correlation based on individual Mann-Whitney tests.

2. Foamy Mϕ

ANOVA - Foamy Mφ

Cases	Sum of Squares	df	Mean Square	F	p
Group	3.242	3	1.081	4.503	.039
Residuals	1.920	8	0.240		

Note. Type III Sum of Squares

Kruskal-Wallis Test

					95% CI for Rank ε^2	
Factor	Statistic	df	p	Rank ε^2	Lower	Upper
Group	9.180	3	.027	0.835	0.831	0.978

Dunn's Post Hoc Comparisons - Group

Comparison	z	W_i	W_j	r_{rb}	p	p_{Bonf}	p_{Holm}
Control - Anti-CTLA-4	-0.801	2.333	4.667	0.778	.423	1.000	.846
Control - Anti-PD-1	-2.346	2.333	9.167	1.000	.019	.114	.095
Control - Combination	-2.575	2.333	9.833	1.000	.010	.060	.060
Anti-CTLA-4 - Anti-PD-1	-1.545	4.667	9.167	1.000	.122	.734	.367
Anti-CTLA-4 - Combination	-1.774	4.667	9.833	1.000	.076	.457	.304
Anti-PD-1 - Combination	-0.229	9.167	9.833	0.222	.819	1.000	.846

Note. Rank-biserial correlation based on individual Mann-Whitney tests.

3. LD Area

ANOVA - LD Area					
**Cases**	Sum of Squares	df	Mean Square	F	p
**Group**	108.7	3	36.23	11.62	.003
**Residuals**	24.93	8	3.117		
Note. **Type III Sum of Squares**					

Kruskal-Wallis Test						
					95% CI for Rank ε^2	
**Factor**	Statistic	df	p	Rank ε^2	Lower	Upper
**Group**	7.667	3	.053	0.697	0.585	0.968

Dunn's Post Hoc Comparisons - Group							
**Comparison**	z	W_i	W_j	r_{rb}	p	p_{Bonf}	p_{Holm}
**Control - Anti-CTLA-4**	2.265	11.00	4.333	-1.000	.024	.141	.118
**Control - Anti-PD-1**	1.359	11.00	7.000	-1.000	.174	1.000	.697
**Control - Combination**	2.491	11.00	3.667	-1.000	.013	.076	.076
**Anti-CTLA-4 – Anti-PD-1**	-0.906	4.333	7.000	0.556	.365	1.000	.773
**Anti-CTLA-4 - Combination**	0.226	4.333	3.667	-0.111	.821	1.000	.821
**Anti-PD-1 - Combination**	1.132	7.000	3.667	-0.778	.258	1.000	.773
Note. **Rank-biserial correlation based on individual Mann-Whitney tests.**							

4. Cell Death Area

ANOVA - Cell Death Area					
**Cases**	Sum of Squares	df	Mean Square	F	p
**Group**	328.5	3	109.5	8.725	.007
**Residuals**	100.4	8	12.55		
Note. **Type III Sum of Squares**					

Kruskal-Wallis Test

					95% CI for Rank ε^2	
**Factor**	Statistic	df	p	Rank ε^2	Lower	Upper
**Group**	8.054	3	.045	0.732	0.561	0.978

Dunn's Post Hoc Comparisons - Group

**Comparison**	z	W_i	W_j	r_{rb}	p	p_{Bonf}	p_{Holm}
**Control - Anti-CTLA-4**	-2.836	2.333	10.67	1.000	.005	.027	.027
**Control - Anti-PD-1**	-1.361	2.333	6.333	0.778	.173	1.000	.702
**Control - Combination**	-1.475	2.333	6.667	1.000	.140	.842	.702
**Anti-CTLA-4 - Anti-PD-1**	1.475	10.67	6.333	-0.778	.140	.842	.702
**Anti-CTLA-4 - Combination**	1.361	10.67	6.667	-1.000	.173	1.000	.702
**Anti-PD-1 - Combination**	-0.113	6.333	6.667	0.111	.910	1.000	.910

Note. **Rank-biserial correlation based on individual Mann-Whitney tests.**
